# Regio- and stereoselective synthesis of new diaminocyclopentanols

**DOI:** 10.3762/bjoc.10.262

**Published:** 2014-10-28

**Authors:** Evgeni A Larin, Valeri S Kochubei, Yuri M Atroshchenko

**Affiliations:** 1 Organic Synthesis Department, Asinex Corporation, 101 North Chestnut, Winston-Salem 27101, NC, USA; 2 Organic Chemistry and Biochemistry Department, Tolstoi State Pedagogical University, 126 Lenin, Tula 300026, Russian Federation

**Keywords:** diaminocyclopentanols, epoxides, regioselectivity, ring opening, stereoselectivity

## Abstract

The optimal conditions for regio- and stereoselective epoxide ring opening of *N,N*-disubstituted 1,2-epoxy-3-aminocyclopentanes by different nucleophilic reagents have been developed. The substituents on the nitrogen atom in the epoxide precursor and the orientation of the oxirane ring are crucial for the reaction outcome. Thus, treatment of (1*RS*,2*SR*,3*SR*)-1,2-epoxy-3-(*N,N*-dibenzylamino)cyclopentane (**3b**) with amines gave a mixture of C1 and C2 regioadducts, while the use of (1*RS*,2*SR*,3*SR*)-1,2-epoxy-3-(*N*-benzyl-*N*-methylamino)cyclopentane (**3a**) led ultimately to C1 adducts. Base-catalyzed aminolysis of epoxides **6a,b** afforded mainly C1 adducts **13a,b** arising from *trans*-diaxal opening of the epoxide ring. Using a Lewis acid catalyst, epoxides **6a,b** were transformed into diaminocyclopentanols **14a,b** via an alternative pathway involving the formation of aziridinium intermediate **17**.

## Introduction

In recent years mimicry of aminoglycosides [1–7] and nucleosides [8–10] has become an important field in pharmaceutical research. Regio- and stereochemical diversities within a sugar-like moiety in those mimics may subtly influence their biological activity [11–14]. The functionalization of synthetic, unnatural aminocyclitols represents an attractive strategy towards the preparation of aminoglycoside and nucleoside mimics, and the development of common synthesis routes to various regio- and stereoisomeric aminocyclitol derivatives remains in demand. One of the optimal routes involves the stereoselective ring opening of epoxides by different nucleophiles in the presence of a variety of activators [15–19]. In this context, epoxidation of cyclic allylic amines and subsequent oxirane ring opening represent a viable approach for the development of new pharmaceutically relevant scaffolds.

As a part of our ongoing research in the development of new aminocyclitols, we exploited cyclopentane derivatives to mimic both the 2-deoxystreptamine ring, a core component in aminoglycosides [7], and nucleosides containing 9*H*-purin-6-amine as a nucleobase portion. High levels of stereoselectivity have been observed in substrate-controlled diastereoselective epoxidation of cyclic alkenes with *O*- and *N*-allylic directing groups [20–21]. Several 3-substituted diastereomeric epoxides have recently been synthesized via the ammonium-directed olefinic oxidation of cyclic allylic amines. It has been reported that functionalization of a range of allylic 3-(*N,N*-dibenzylamino)cycloalkenes with *m*-CPBA in the presence of trichloroacetic acid gave exclusively corresponding *syn*-epoxides [22]. Examples of stereoselective epoxide opening of these cyclic amine derivatives are limited to the preparation of the corresponding diols under acidic conditions [23]. Other reported strategies involve the formation of diaminocyclohexanols from epoxides under basic conditions [24] or by activating the epoxides with hydrogen bond donors [25]. Additionally, the synthesis of aminocyclitols from cyclitol epoxides has been described [26–27]. It has been shown that the reaction of cyclitol epoxides with nitrogen-containing nucleophiles in the presence of Lewis acids gave a mixture of C1 and C2 adducts. Both epoxide carbons can react with a nucleophile to produce regioisomeric aminocyclitols. Herein, we describe the regio- and stereoselective synthesis of diaminocyclopentanol derivatives from *N*-protected cyclopentanamine epoxides using nitrogen-containing nucleophiles.

## Results and Discussion

### Preparation of starting epoxides

**Scheme 1:**
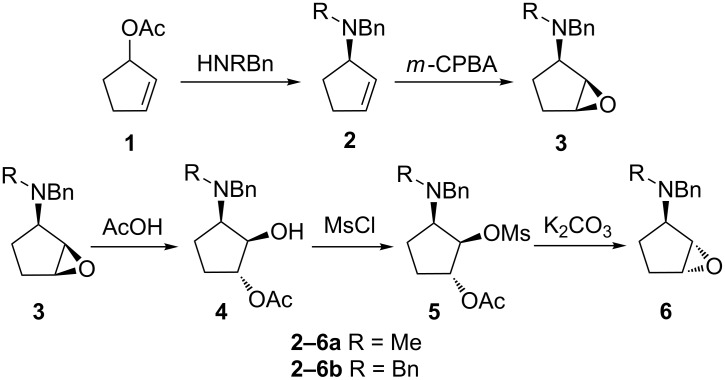
Preparation of the starting materials.

Epoxides **3a,b** (Scheme 1) were obtained by the addition of benzyl(methyl)amine or dibenzylamine to cyclopent-2-en-1-yl acetate (**1**) followed by epoxidation [28]. Epoxides **6a,b** were synthesized from **3a,b** through epoxide ring inversion using glacial acetic acid as the oxirane-cleaving agent [29].

**Figure 1:**
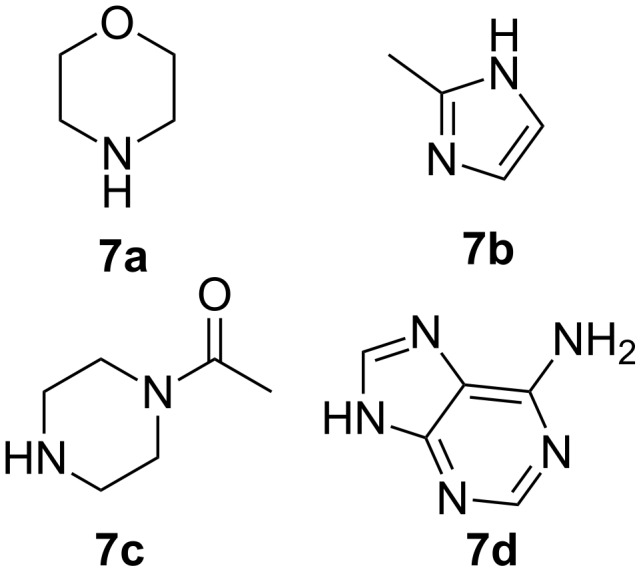
Amine-based nucleophiles used in the epoxide ring opening reaction.

The treatment of the corresponding acetates **4** with mesyl chloride and subsequent transesterification of mesylated substrates **5** resulted in the formation of **6a,b**. Epoxides **3** and **6** were identified by ^1^H NMR data [28]. Morpholine (**7a**), 2-methyl-1*H*-imidazole (**7b**), *N*-acetylpiperazine (**7c**) and 9*H*-purin-6-amine (**7d**) were used as nucleophiles (Figure 1). Starting amines were selected based on the fact that these motifs are common structural features in drug molecules.

### Optimization of the epoxide ring opening reaction of **3a**

The opening of epoxides with nucleophiles in the presence of Lewis acid or base promoters is well documented [30–34]. We conducted a number of experiments to optimize the ring opening in **3a** (Table 1). The initial catalytic epoxide ring-opening experiments of **3a** in MeCN at 80 °C [35] were unsuccessful, since only starting material was recovered. A series of experiments was performed under solvent-free conditions at 100 °C. In case of morpholine (**7a**), the best catalytic effect was observed with LiClO_4_ [36] and Zn(ClO_4_)_2_·6H_2_O [37] affording 56 and 76% yield of **8a** after isolation and purification, therefore the absence of the solvent seems crucial for the reaction outcome (Table 1, entries 2 and 3).

**Table 1:** Reactions of **3a** with nucleophiles in the presence of various catalysts.

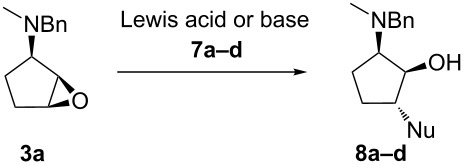					

Entry	Nu	Catalyst	**8**	Yield (%)^a,b^	Yield (%)^b,c^

1	**7a**	ZrCl_4_	**8a**	14^d^	traces
2	**7a**	LiClO_4_	**8a**	56	–
3	**7a**	Zn(ClO_4_)_2_·6H_2_O	**8a**	76	5
4	**7a**	Bi(OTf)_3_	**8a**	5	traces
5	**7a**	Cs_2_CO_3_	**8a**	–	traces
6	**7a**	K_2_CO_3_	**8a**	–	-–
7	**7b**	ZrCl_4_	**8b**	traces^d^	traces^d^
8	**7b**	LiClO_4_	**8b**	27	–
9	**7b**	Zn(ClO_4_)_2_·6H_2_O	**8b**	21	–
10	**7b**	Bi(OTf)_3_	**8b**	53	24^d^
11	**7b**	Cs_2_CO_3_	**8b**	61	75
12	**7b**	K_2_CO_3_	**8b**	38	16^d^
13	**7c**	Zn(ClO_4_)_2_·6H_2_O	**8c**	44	7
14	**7d**	Cs_2_CO_3_	**8d**	–	65

^a^Reagents and conditions: 5.0 mmol epoxide, 10.0 mol % catalyst, 6.5 mmol nucleophile, neat, 100 °C, 2 h. ^b^Isolated yield. ^c^Reagents and conditions: 5.0 mmol epoxide, 10.0 mol % catalyst, 6.5 mmol nucleophile, DMSO (10 mL), 120 °C, 2 h. ^d^Reaction time 4 h.

On the contrary, the epoxide ring opening of **3a** with 2-methyl-1*H*-imidazole (**7b**) in the presence of Lewis acid catalysts produced **8b** in low yields (Table 1, entries 7–9), except for the reaction in the presence of Bi(OTf)_3_ [38] (Table 1, entry 10). In this case, we planned to evaluate the catalytic efficiency of base promoters such as Cs_2_CO_3_ and K_2_CO_3_ [39–41]. The best result was obtained with Cs_2_CO_3_ using DMSO as a solvent (Table 1, entry 11). The catalytic effect of Cs_2_CO_3_ could be explained by its ability to increase the poor nucleophilicity of 2-methyl-1*H*-imidazole (**7b**). Eventually, Zn(ClO_4_)_2_·6H_2_O and Cs_2_CO_3_ were selected as the catalysts for further experiments. These conditions were applied for aminolysis of **3a** with *N*-acetylpiperazine (**7c**) and 9*H*-purin-6-amine (**7d**) (Table 1, entries 13 and 14) to provide 44 and 65% yields of the corresponding aminocyclopentanols **8c** and **8d** (Supporting Information File 1).

In every experiment, 1,2-*trans*-2,3-*cis*-aminocyclopentanols, arising from opening of epoxide **3a** at C1, were the only regioisomers isolated. The stereo- and regiochemistry of **8a** and **8d** were assigned by 2D NMR (HSQC-DEPT, ^1^H,^1^H COSY and NOESY experiments). The 2D carbon–proton chemical shift correlation study on **8a** showed that the proton resonances at δ 2.43–2.46, 2.55 and 3.90 correspond to methylene groups, and that these resonances can be assigned by C(1)H-OH and C(2)H-NCH_3_ COSY correlations. The presence of the C(1)H-C(2)H correlation and the absence of the C(2)H-C(5)H correlation in the NOESY spectrum support the stereochemistry of **8a**. The same relative configuration of **8d** was assigned from 2D NMR analysis, and the stereochemistry of **8b** and **8c** was additionally confirmed by the comparison of ^3^*J* coupling constants of the resonances corresponding to C(1)H, C(2)H and C(5)H (Supporting Information File 2). Therefore, spectral data obtained for the compounds **8a–d** are consistent with the acid-catalyzed *trans*-diaxial epoxide opening, proceeding via a late-transition state, and the *N*-benzyl-*N*-methylammonium moiety promotes the nucleophilic attack at the C1-oxirane carbon atom [29].

### Synthesis of diaminocyclopentanols using epoxide **3b**

Next, we explored the influence of the *N*,*N*-dibenzylamino group on the ring opening reaction using the optimized reaction conditions for **3b** (Table 2). Surprisingly, the ring opening of *N*,*N*-dibenzyl derivative **4b** displayed poor regioselectivity. In fact, a mixture of the separable regioisomers **9a–d** and **10a–d** were obtained, where the major products **9a–d** were formed due to the attack of the nucleophile at the C1-oxirane carbon atom.

**Table 2:** Regioselectivity in the epoxide ring opening of **3b** with nucleophiles.

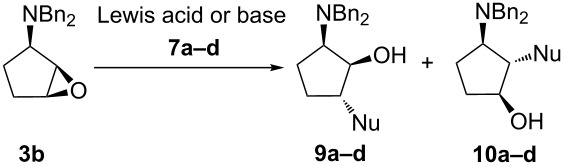					

Entry	Nu	Catalyst	Product	Yield (%)^a,b^	rr^c^ (**9**:**10**)

1	**7a**	Zn(ClO_4_)_2_·6H_2_O	**9a**	48	2:1
			**10a**	24	
2	**7a**	LiClO_4_	**9a**	50	8:5
			**10a**	31	
3	**7b**	Cs_2_CO_3_	**9b**	47	7:3
			**10b**	20	
4	**7b**	Cs_2_CO_3_	**9b**	64^d^	2:1
			**10b**	26^d^	
5	**7c**	Zn(ClO_4_)_2_·6H_2_O	**9c**	43	2:1
			**10c**	21	
6	**7c**	LiClO_4_	**9c**	45^d^	9:5
			**10c**	25^d^	
7	**7d**	Cs_2_CO_3_	**9d**	46	2:1
			**10d**	23	

^a^Reagents and conditions: 5.0 mmol epoxide, 10.0 mol % catalyst, 6.5 mmol nucleophile, neat, 100 °C, 2 h. ^b^Isolated yield. ^c^Regioisomeric ratio for separated isomers. ^d^Reactions were performed in DMSO at 120 °C.

In case of aliphatic cyclic amines (morpholine (**7a**) and *N*-acetylpiperazine (**7c**)), the best regioisomeric ratio (2:1) was observed using Zn(ClO_4_)_2_·6H_2_O as a catalyst (Table 2, entries 1 and 5). The use of LiClO_4_ led to lower regioselectivity (Table 2, entries 2 and 6). Apparently, the nature of the catalyst and the ability of the metal ion to coordinate with the oxirane oxygen have no significant influence on the regioisomeric ratio (rr). The reactions of **3b** with 2-methyl-1*H*-imidazole (**7b**) and 9*H*-purin-6-amine (**7d**) in the presence of Cs_2_CO_3_ in DMSO showed the same regioselectivity (Table 2, entries 4 and 7), indicating that the epoxide opening reactions are not directed by the amine nucleophilicity. Moreover, there was not much effect on the outcome of the reactions conducted either under solvent-free conditions or using DMSO as a solvent.

The regioisomers **9a–d** and **10a–d** were isolated through column chromatographic separation and fully characterized in order to avoid ambiguity. The stereochemistry of the major regioisomers **9a–d** was deduced from the analysis of ^1^H NMR and 2D NMR data as described for **8a–d**. Assignments of CH proton resonances of **10a–d** were established by ^1^H,^13^C HMBC and HSQC-DEPT experiments, and connectivity was established by the analysis of ^1^H,^1^H COSY spectra. The structures of **10a** and **10c** were elucidated based on COSY correlation of C(1)H-OH resonances at δ 3.88 for **10a** and 3.85 for **10c**. ^1^H NMR NOESY analyses of **10a** and **10c** facilitated the initial assignment of the relative configuration. Thus, the values of C(1)H-C(3)H, C(2)H-OH and C(3)H-CH_2_N NOEs were quite diagnostic. C(1)H proton resonances were observed at δ 3.84–3.95 for **10b** and 4.53 for **10d**. C(2)H and C(3)H resonances appeared as doublets of doublets at δ 4.20 (*J* = 9.3 and 7.8 Hz) and 3.38 (*J =* 17.7 and 8.6 Hz) for **10b** and at δ 4.61 (*J =* 9.8 and 7.9 Hz) and 3.71 (*J =* 18.3 and 8.7 Hz) for **10d**. C(2)H-C(3)H, C(1)H-OH, C(2)H-C(1)H COSY correlations and C(1)H-C(3)H, C(2)H-OH, C(2)H-CH_2_Ph NOEs indicated the relative 1,2-*anti*-2,3-*anti*-configurations of **10b** and **10d** (Supporting Information File 2).

In order to investigate the influence of different substituents on the nitrogen atom on the regioselective outcome, epoxides **3c** and **3d** were additionally synthesized, and the results are summarized in Table 3.

**Table 3:** Epoxide ring opening of **3a–d** containing different substituents on the nitrogen atom.

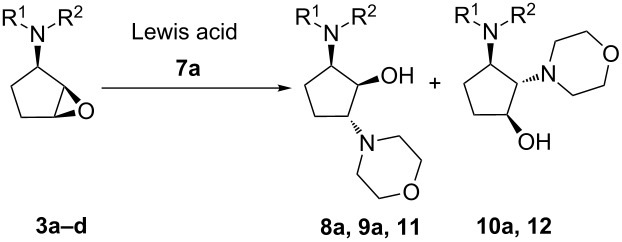							

Entry	Epoxide	R^1^	R^2^	Reaction time, h^a^	Product (C1)^b^	Product (C2)^b^	rr (C1:C2)

1	**3a**	Me	Bn	2	**8a**	–	>19:1
2	**3b**	Bn	Bn	2	**9a**	**10a**	2:1
3	**3c**	Ph	Bn	6	**11**	**12**	5:1^c^
4	**3d**	Ph	Ph	6^d^	–	–	–

^a^Reagents and conditions: 5.0 mmol epoxide, 10.0 mol % Zn(ClO_4_)_2_·6H_2_O, 6.5 mmol morpholine (**7a**), neat, 100 °C. ^b^Products formed due to the nucleophilic attack at the C1 or C2 oxirane carbon atoms. ^c^The regiochemistry was established from ^1^H NMR analysis of the mixture. ^d^No reaction was observed.

It has been proposed earlier that the coordination of the Lewis acid to both oxygen atoms in 2,3-epoxy alcohols and acids leads to the formation of the intermediate complex, for which nucleophiles attack preferably the C3 position [42–43]. The electron donating methyl group in **3a** seems to improve the binding of the Lewis acid to the nitrogen atom, favoring the formation of the C1-adduct, and the lower basicity of the dibenzylamino moiety in **3b** may lead to the diminished coordination of Lewis acid and hence the lower regioselectivity (Table 3, entries 1 and 2). Unexpectedly, using **3c**, with an electron withdrawing phenyl group on the nitrogen atom, provided higher regioselectivity towards the C1-adduct (rr 5:1) in comparison with **3b** (Table 3, entries 2 and 3). The structure of the major regioisomer **11** was established by the analysis of ^1^H NMR and ^1^H,^1^H COSY data (Supporting Information File 2). This fact can be explained by the suggestion that despite the induced binding of the Lewis acid to the nitrogen atom due to the negative inductive effect of the phenyl substituent, the intermediate complex is likely to be stabilized by the π electrons of the phenyl ring, which leads to the formation of **11** as a major product. However, the presence of electron withdrawing substituents on the nitrogen atom and, as a result, the diminished coordination of the Lewis acid required the longer reaction time (6 h), while in case of **3d** bearing two phenyl groups no epoxide ring opening was observed (Table 3, entries 3 and 4).

### Ring opening reactions for epoxides **6a,b**

Ring opening of epoxides **6a,b** was investigated by the reaction with 9*H*-purin-6-amine (**7d**) and morpholine (**7a**) as the nucleophiles under the above conditions (Table 4). As it was expected, single regioisomers **13a,b** with 1,2-*anti*-2,3-*anti*-configuration (Table 4, entries 1 and 2) were obtained. C(1)H-C(2)H, C(1)H-OH, C(1)H-C(5)H COSY correlations and C(2)H-C(5)H, C(2)H-OH, C(5)H-OH NOEs demonstrated that product **13b** has the structure shown in Table 4. The presence of C(2)H-C(5)H and C(1)H-NCH_3_ NOEs were supportive of the assigned configuration of **13a** (Supporting Information File 2). The ring opening reaction of epoxide **6b** with 9*H*-purin-6-amine (**7d**) in the presence of Cs_2_CO_3_ (Table 4, entry 2) showed the higher level of regioselectivity in comparison with the regioselective outcome for epoxide **3b** (Table 2, entry 7), which can be interpreted as a result of dominance of steric over electronic factors in the case of epoxides **6a,b**.

**Table 4:** The epoxide ring opening reactions of **6a,b**.

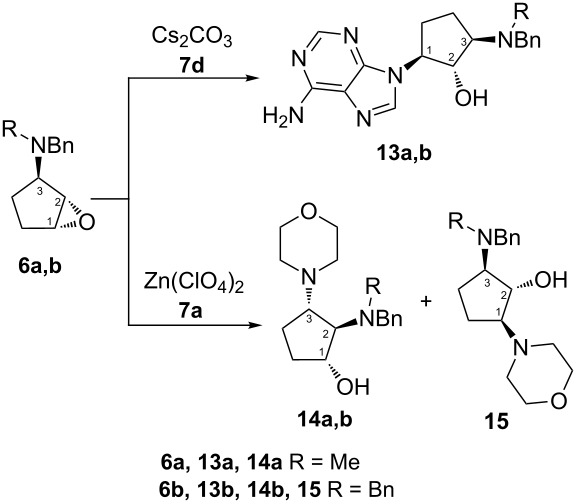					

Entry	Epoxide	Nu	Catalyst	Product	Yield (%)^a^

1	**6a**	**7d**	Cs_2_CO_3_	**13a**	68^b^
2	**6b**	**7d**	Cs_2_CO_3_	**13b**	55^b^
3	**6a**	**7a**	Zn(ClO_4_)_2_·6H_2_O	**14a**	84^c^
4	**6b**	**7a**	Zn(ClO_4_)_2_·6H_2_O	**14b**	80^c^
				**15**	6^d^

^a^Isolated yield. ^b^Reagents and conditions: 5.0 mmol epoxide, 10.0 mol % catalyst, 6.5 mmol nucleophile, DMSO (10 mL), 120 °C, 2 h. ^c^Reagents and conditions: 5.0 mmol epoxide, 10.0 mol % catalyst, 6.5 mmol nucleophile, neat, 100 °C, 2 h. ^d^Regioisomeric ratio (**14b**:**15**) – 12:1.

A bulky *N*,*N*-disubstituted amino group is prone to adopt a pseudoequatorial orientation. In basic conditions, the nucleophile (9*H*-purin-6-amine (**7d**)) attacks the oxirane carbon atom from the side of the carbocyclic ring where the *N*,*N*-disubstituted amino group is located, and this precludes the approach of the nucleophile to the C2 carbon atom because of sterical hindrance. Thus, C1 of **6a,b** is the favoured site for the nucleophilic attack, which gives rise to the formation of products **13a**,**b** with essentially complete regioselectivity (Table 4, entries 1 and 2). Surprisingly, aminolysis of substrates **6a,b** under Lewis acid-catalyzed conditions resulted mostly in the formation of regioisomers **14a,b** (Table 4, entries 3 and 4), while the target isomer **15** was obtained only from epoxide **6b,** as the minor product in 6% yield (Table 4, entry 4). Aminocyclopentanols **14a,b** provided quite similar ^1^H NMR spectra, and methine protons showed similar multiplicity patterns. For example, the resonance corresponding to C(2)H of **14a** appeared as a doublet of doublets (*J* = 7.0 and 4.4 Hz) centered at δ 2.84, while the signal corresponding to C(2)H of **14b** also appeared as a doublet of doublets (*J* = 7.3 and 4.3 Hz) centered at δ 2.92. The structure and the relative configuration of **14a,b** were unambiguously confirmed by the presence of C(1)H-C(2)H, C(1)H-OH, C(2)H-C(3)H COSY and C(1)H-C(3)H, C(2)H-OH, C(3)H-CH_2_Ph NOESY correlations observed in 2D spectra. The structure of **15** was determined by the analysis of 2D NMR spectra by analogy with that of **13b** (Supporting Information File 2).

**Scheme 2:**
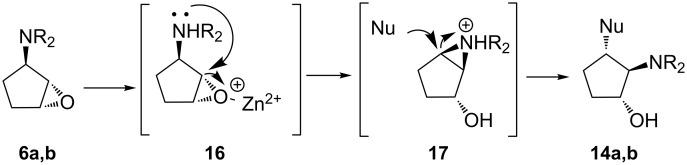
Postulated mechanism for the formation of **14a,b**.

These results are in contrast to the outcome of the ring opening reactions of epoxides **3a,b**, and this may be explained by the formation of **14a,b** via the aziridimium intermediate **17**. Based on earlier results [44], a mechanism of this transformation was hypothesized as shown in Scheme 2. The intermediate **17** is formed after the intramolecular rearrangement of intermediate **16** formed from Zn(ClO_4_)_2_-catalyzed C–O bond cleavage followed by the attack of the *N*,*N*-disubstituted amino moiety towards C2. The approach of the *N*,*N*-disubstituted amino group to C2 would be more favorable than that of the nucleophile (morpholine) to either oxirane carbon atoms. Therefore, the nucleophilic attack is subsequent to the formation of the aziridinium ring, which is consistent with our experimental results.

## Conclusion

In summary, we have optimized the reaction conditions of epoxide ring opening of epoxides **3a,b** and **6a,b** with a variety of amines to give the corresponding diaminocyclopentanols in good yields. It has been shown that using Zn(ClO_4_)_2_·6H_2_O under solvent-free conditions and Cs_2_CO_3_ in DMSO is preferable to the ring opening of di-*N*-protected cyclopentanamine epoxides. We have highlighted the influence of the nature of the *N*,*N*-disubstituted amino moiety and the orientation of the oxirane ring on the stereo- and regioselective outcomes. Aminolysis of epoxides **3a,b** is mainly dictated by electronic bias to afford the corresponding C1 adducts for **3a** and the mixture of C1 and C2 adducts in the ratio 2:1 for **3b**. The treatment of epoxides **6a,b** with 9*H*-purin-6-amine (**7d**) under base-catalyzed conditions gives C1 adducts as the sole products. Thus, the nucleophilic attack of the amine towards the C2 oxirane carbon atom can be controlled by steric constraints, and it is obvious that the bulky *N*,*N*-dibenzylamino moiety of epoxide **6b** impedes the formation of the corresponding C2 adduct due to the steric hindrance. Application of Lewis acid as a catalyst for the ring opening reactions of **6a,b** provides an alternative mechanism that involves the formation of aziridinium intermediate **17**. As a result, regioisomers **14a,b** were obtained as the major products.

## Supporting Information

File 1 Experimental and characterization data.

File 2 Copies of ^1^H and ^13^C NMR spectra.
