# Supplementary material for: Regio- and stereoselective synthesis of new diaminocyclopentanols
Source: Beilstein J Org Chem. 2014 Oct 28;10:2513–20. doi: 10.3762/bjoc.10.262 (PMC4222406; doi:10.3762/bjoc.10.262)
Supplement: File 1 — Experimental and characterization data. [file Beilstein_J_Org_Chem-10-2513-s001.pdf]

Supporting Information

for

Regio- and stereoselective synthesis of new diaminocyclopentanols

Evgeni A. Larin*[1], Valeri S. Kochubei[1] and Yuri M. Atroshchenko[2]

Address: [1]Organic Synthesis Department, Asinex Corporation, 101 North Chestnut, Winston-Salem 27101, NC, USA and [2]Organic Chemistry and Biochemistry Department, Tolstoi State Pedagogical University, 126 Lenin, Tula 300026, Russian Federation

Email: Evgeni A. Larin - elarin@asinex.com

* Corresponding author

Experimental and characterization data

Table of contents

General information S2

Experimental details and characterization data S3–S8

References S9

General information

^{1}H, ^{13}C and the detailed 2D NMR spectra were determined on Varian Mercury Plus 400 spectrometer. Chemical shifts are reported in parts per million using solvent as the internal standard ($CDCl_3$, DMSO-d_6 or C_5D_5N). LRMS was run on Finnigan Surveyor MSQ mass spectrometer. HRMS spectra were obtained on Bruker MicrOTOF II mass spectrometer. Thin layer chromatography was performed on DC-Alufoilen Kieselgel $60F_{254}$ 0.2 mm plates (Merck) and visualized under UV light and stained with ninhydrin or Seebach solution followed by heating. Column chromatography was done by using Kieselgel 60 (Merck) 60–200 mesh as the stationary phase. All reagents and solvents were purchased from commercial sources and used without further purification. Starting epoxides **3a,b** and **6** previously described in the literature[1,2] were characterized by comparing their ^{1}H NMR spectra to the published data. All yields reported in this publication refer to isolated ones of compounds and their purity was determined by ^{1}H NMR. The stereochemistry displayed in the products is relative and not absolute.

General procedure for Lewis acid-catalyzed ring opening reactions of epoxides 3 and 6 as examplified by 8a

(1*RS*,2*RS*,5*RS*)-2-[Benzyl(methyl)amino]-5-(morpholin-4-yl)cyclopentanol (**8a**)

To the mixture of **3a** (1.02 g, 5 mmol) and morpholine (0.57 g, 6.5 mmol) zinc(II) perchlorate hexahydrate (0.19 g, 10 mol%) was added, and the mixture was magnetically stirred at 100 °C for 2 h under nitrogen atmosphere. After completion of the reaction (disappearance of starting material monitored by TLC), the reaction mixture was diluted with CH_2Cl_2 (30 mL) and filtered through celite. The filtrate was washed with water (3×30 mL), dried over anhydrous Na_2SO_4 and concentrated *in vacuo* to give the crude product, which was purified by flash chromatography (35% EtOAc/hexane) to give the title compound **8a** (1.10 g, 76%) as a colourless oil; d_H (400 MHz, DMSO-d_6) 7.34-7.18 (5H, m, Ph), 4.05 (1H, s, OH), 3.90 (1H, d, *J* 4.9 Hz, H-1), 3.64 (1H, d, *J* 13.3 Hz, CH_2Ph), 3.55 (4H, t, *J* 4.6 Hz, CH_2O), 3.47 (1H, d, *J* 13.3 Hz, CH_2Ph), 2.55 (1H, dt, *J* 12.2, 6.1 Hz, H-2), 2.50-2.47 (2H, m, CH_2N), 2.46-2.43 (1H, m, H-5), 2.39-2.32 (2H, m, CH_2N), 2.10 (3H, s, CH_3), 1.88-1.75 (2H, m, H-3 and H-4), 1.64-1.51 (1H, m, H-3), 1.38-1.25 (1H, m, H-4); d_C (100 MHz, DMSO-d_6, HSQC-DEPT) 128.2, 127.8, 126.3 (Ph), 73.8 (C-5), 71.2 (C-1), 67.4 (C-2), 65.9 (CH_2O), 59.0 (CH_2Ph), 51.3 (CH_2N), 39.2 (CH_3), 26.4 (C-3), 25.5 (C-4); LC-MS (APCI): *m/z* = 291.13 $[M]^+$. HRMS (ESI): *m/z* calcd for $[C_{17}H_{26}N_2O_2]^+$: 291.2067; found: 291.2071..

(1*RS*,2*RS*,5*RS*)-2-(4-Acetylpiperazin-1-yl)-5-[benzyl(methyl)amino]cyclopentanol (**8c**)

This compound was isolated in 44% yield (0.73 g) as a pale yellow oil; d_H (400 MHz, DMSO-d_6) 7.33-7.18 (5H, m, Ph), 4.08 (1H, s, OH), 3.90 (1H, d, *J* 5.0 Hz, H-1), 3.64 (1H, d, *J* 13.3 Hz, CH_2Ph), 3.47 (1H, d, *J* 13.3 Hz, CH_2Ph), 3.42-3.34 (4H, m, CH_2N), 2.59-2.51 (1H, m, H-2), 2.49-2.46 (1H, m, H-5), 2.44-2.25 (4H, m, CH_2N), 2.10 (3H, s, NCH_3), 1.96 (3H, s, $OCCH_3$), 1.90-1.75 (2H, m, CH_2), 1.63-1.50 (1H, m, CH_2), 1.39-1.28 (1H, m, CH_2); d_C (100 MHz, DMSO-d_6) 167.8, 138.9, 128.5, 127.9, 126.6, 73.6, 71.6, 67.6, 59.2, 51.1, 50.5, 45.6, 40.8, 26.5, 26.0; LC-MS (APCI): *m/z* = 332.17 $[M]^+$. HRMS (ESI): *m/z* calcd for $[C_{19}H_{29}N_3O_2]^+$: 332.2333; found: 332.2334.

(1*RS*,2*RS*,5*RS*)-2-(Dibenzylamino)-5-(morpholin-4-yl)cyclopentanol (**9a**)

This compound was isolated in 48% yield (0.88 g) as a white solid, and regioisomer **10a** was obtained in 24% yield (0.44 g) as a white solid; *Compound **9a***: d_H (400 MHz, DMSO-d_6) 7.35-7.15 (10H, m, Ph), 4.25 (1H, d, *J* 3.1 Hz, OH), 3.87 (1H, s, H-1), 3.72 (4H, s, CH_2Ph), 3.51 (4H, t, *J* 4.6 Hz, CH_2O), 2.87-2.80 (1H, m, H-2), 2.46-2.42 (2H, m, CH_2N), 2.46-2.43 (1H, buried m, H-5), 2.34-2.26 (2H, m, CH_2N), 1.79-1.71 (1H, m, H-4), 1.69-1.63 (1H, m, H-3), 1.61-1.51 (1H, m, H-3), 1.22-1.10 (1H, m, H-4); d_C (100 MHz, DMSO-d_6, HSQC-DEPT) 128.3, 127.9, 127.0 (Ph), 74.1 (C-5), 72.6 (C-1), 66.9 (CH_2O), 66.1 (C-2), 55.9 (CH_2Ph), 52.4 (CH_2N), 26.7 (C-3), 26.6 (C-4); LC-MS (APCI): *m/z* = 367.22 $[M]^+$. HRMS (ESI): *m/z* calcd for $[C_{23}H_{30}N_2O_2]^+$: 367.2380; found: 367.2374. *Compound **10a***: d_H (400 MHz, DMSO-d_6) 7.39-7.15 (10H, m, Ph), 4.42 (1H, d, *J* 4.8 Hz , OH), 3.88 (1H, dd, *J* 10.0, 5.0 Hz, H-1), 3.78 (2H, d, *J* 13.8 Hz, CH_2Ph), 3.44 (2H, br s, CH_2O), 3.42 (2H, buried d, *J* 13.8 Hz, CH_2Ph), 3.19 (2H, s, CH_2O), 3.02 (1H, q, *J* 7.8 Hz, H-3), 2.60 (1H, dd, *J* 7.1, 4.7 Hz, H-2), 2.46-2.39 (2H, m, CH_2N), 2.38-2.30 (2H, m, CH_2N), 1.81-1.69 (1H, m, H-4), 1.61-1.45 (3H, m, H-4 and H-5); d_C (100 MHz, DMSO-d_6, HSQC-DEPT) 128.1, 127.4, 126.2 (Ph), 74.5 (C-2), 70.4 (C-1), 60.1 (C-3), 53.8 (CH_2O), 53.8 (CH_2Ph), 50.7 (CH_2N), 32.0 (C-4), 20.2 (C-5); LC-MS (APCI): *m/z* = 367.31 $[M]^+$. HRMS (ESI): *m/z* calcd for $[C_{23}H_{30}N_2O_2]^+$: 367.2380; found: 367.2374.

(1*RS*,2*RS*,5*RS*)-2-(4-Acetylpiperazin-1-yl)-5-(dibenzylamino)cyclopentanol (**9c**)

This compound was isolated in 43% yield (0.87 g) as a white solid, and regioisomer **10c** was obtained in 21% yield (0.43 g) as a white solid; *Compound **9c***: d_H (400 MHz, DMSO-d_6) 7.35-7.14 (10H, m, Ph), 4.25 (1H, d, *J* 26.5 Hz, OH), 3.88 (1H, dd, *J* 6.0, 2.5 Hz, H-1), 3.72 (4H, s, CH_2Ph), 3.44 (1H, dd, *J* 14.0, 7.1 Hz, H-2), 3.36 (4H, s, CH_2N), 2.89-2.79 (1H, m, H-5), 2.50-2.21 (4H, m, CH_2N), 1.95 (3H, s, $OCCH_3$), 1.78-1.72 (1H, m, CH_2), 1.71-1.63 (1H, m, CH_2), 1.62-1.50 (1H, m, CH_2), 1.23-1.11 (1H, m, CH_2); d_C (100 MHz, DMSO-d_6) 167.8, 139.9, 128.2, 127.9, 126.4, 73.4, 72.5, 64.2, 55.5, 51.1, 45.5, 40.8, 25.6, 25.4, 20.8; LC-MS (APCI): *m/z* = 408.22 $[M]^+$. HRMS (ESI): *m/z* calcd for $[C_{25}H_{33}N_3O_2]^+$: 408.2646; found: 408.2641. *Compound **10c***: d_H (400 MHz, DMSO-d_6) 7.39-7.14 (10H, m, Ph), 4.45 (1H, s, OH), 3.85 (1H, s, H-1), 3.78 (2H, d, *J* 13.8 Hz, CH_2Ph), 3.41 (2H, d, *J* 13.8 Hz, CH_2Ph), 3.34-3.12 (4H, m, CH_2N), 3.01 (1H, s, H-3), 2.65 (1H, s, H-2), 2.34 (4H, s, CH_2N), 1.94 (3H, s, $OCCH_3$), 1.82-1.68 (1H, m, H-4), 1.62-1.44 (3H, m, H-4 and H-5); d_C (100 MHz, DMSO-d_6,

HSQC-DEPT) 128.1, 127.5, 126.2 (Ph), 73.9 (C-2), 70.5 (C-1), 59.8 (C-3), 53.7 (CH_2Ph), 49.7 (CH_2N), 40.7 (CH_2N), 20.4 (CH_3), 19.9 (C-5), 19.7 (C-4); LC-MS (APCI): *m/z* = 408.32 $[M]^+$. HRMS (ESI): *m/z* calcd for $[C_{25}H_{33}N_3O_2]^+$: 408.2646; found: 408.2651.

(1*RS*,2*RS*,3*SR*)-2-[Benzyl(methyl)amino]-3-(morpholin-4-yl)cyclopentanol (**14a**)

This compound was isolated in 84% yield (1.22 g) as a yellow oil; d_H (400 MHz, DMSO-d_6) 7.33-7.15 (5H, m, Ph), 4.42 (1H, d, *J* 3.1 Hz, OH), 4.11 (1H, s, H-1), 3.63 (2H, dd, *J* 34.8, 13.6 Hz, CH_2Ph), 3.54 (4H, t, *J* 4.6 Hz, CH_2O), 2.84 (1H, dd, *J* 7.0, 4.4 Hz, H-2), 2.68 (1H, dd, *J* 14.7, 7.4 Hz, H-3), 2.54-2.40 (4H, m, CH_2N), 2.15 (3H, s, CH_3), 1.67-1.55 (3H, m, H-4 and H-5), 1.54-1.43 (1H, m, H-5); d_C (100 MHz, DMSO-d_6, HSQC-DEPT) 128.5, 127.7, 126.1 (Ph), 74.1 (C-2), 69.1 (C-1), 66.1 (CH_2O), 66.0 (C-3), 58.1 (CH_2Ph), 50.7 (CH_2N), 37.7 (CH_3), 32.2 (C-4), 23.4 (C-5); LC-MS (APCI): *m/z* = 291.28 $[M]^+$. HRMS (ESI): *m/z* calcd for $[C_{17}H_{26}N_2O_2]^+$: 291.2067; found: 291.2074.

(1*RS*,2*RS*,3*SR*)-2-(Dibenzylamino)-3-(morpholin-4-yl)cyclopentanol (**14b**)

This compound was isolated in 80% yield (1.46 g) as a white solid, and regioisomer **15** was obtained in 6% yield (0.11 g) as a white solid; *Compound **14b***: d_H (400 MHz, DMSO-d_6) 7.40-7.14 (10H, m, Ph), 4.48 (1H, d, *J* 4.5 Hz, OH), 4.27-4.18 (1H, m, H-1), 3.73 (2H, d, *J* 13.7 Hz, CH_2Ph), 3.57 (2H, d, *J* 13.7 Hz, CH_2Ph), 3.47-3.36 (4H, m, CH_2O), 2.92 (1H, dd, *J* 7.3, 4.3 Hz, H-2), 2.71 (1H, q, *J* 7.6 Hz, H-3), 2.27 (4H, t, *J* 4.5 Hz, CH_2N), 1.70-1.60 (1H, m, H-5), 1.59-1.52 (2H, m, H-4), 1.51-1.41 (1H, m, H-5); d_C (100 MHz, DMSO-d_6, HSQC-DEPT) 128.3, 127.4, 126.0 (Ph), 79.5 (C-1), 69.1 (C-2), 66.2 (C-3), 66.0 (CH_2O), 54.1 (CH_2Ph), 50.4 (CH_2N), 32.4 (C-5), 22.8 (C-4); LC-MS (APCI): *m/z* = 367.35 $[M]^+$. HRMS (ESI): *m/z* calcd for $[C_{23}H_{30}N_2O_2]^+$: 367.2380; found: 367.2373. *Compound **15***: d_H (400 MHz, DMSO-d_6) 7.38-7.13 (10H, m, Ph), 4.46 (1H, d, *J* 6.2 Hz, OH), 3.95 (1H, dd, *J* 13.6, 6.8 Hz, H-1), 3.72 (2H, d, *J* 14.2 Hz, CH_2Ph), 3.58 (2H, d, *J* 14.2 Hz, CH_2Ph), 3.53 (4H, t, *J* 4.6 Hz, CH_2O), 2.96 (1H, q, *J* 7.9 Hz, H-2), 2.58-2.49 (2H, m, CH_2N), 2.44-2.36 (2H, m, CH_2N), 2.33 (1H, dd, *J* 14.3, 7.3 Hz, H-5), 1.66-1.40 (4H, m, CH_2); d_C (100 MHz, DMSO-d_6, HSQC-DEPT) 128.0, 127.4, 125.9 (Ph), 74.9 (C-1), 69.6 (C-5), 66.0 (C-2), 65.9 (CH_2O), 53.7 (CH_2Ph), 50.7 (CH_2N), 23.5 (CH_2), 21.5 (CH_2); LC-MS (APCI): *m/z* = 367.26 $[M]^+$. HRMS (ESI): *m/z* calcd for $[C_{23}H_{30}N_2O_2]^+$: 367.2380; found: 367.2378.

General procedure for ring opening reactions of epoxides 3 and 6 under basic conditions as exemplified by 8b

(1*RS*,2*RS*,5*RS*)-2-[Benzyl(methyl)amino]-5-(2-methyl-1*H*-imidazol-1-yl)cyclopentanol (**8b**)

To a solution of **3a** (1.02 g, 5 mmol) and 2-methyl-1*H*-imidazole (0.53 g, 6.5 mmol) in DMSO (10 mL) cesium carbonate (0.16 g, 10 mol %) was added. The mixture was heated at 120 °C for 2 h under vigorous stirring. After completion of the reaction as monitored by TLC, the reaction mixture was diluted with CH_2Cl_2 (30 mL) and water (30 mL). The organic layer was washed with water (3×30 mL), dried over anhydrous Na_2SO_4. The solvent was evaporated *in vacuo* to give the crude product, which was purified by flash chromatography (50% EtOAc/hexane) to give the title compound **8b** (1.07 g, 75%) as a colourless oil; d_H (400 MHz, DMSO-d_6) 7.34-7.18 (5H, m, Ph), 7.02 (1H, d, *J* 0.9 Hz, CH), 6.73 (1H, d, *J* 0.7 Hz, CH), 4.63 (1H, s, OH), 4.35 (1H, td, *J* 7.8, 2.9 Hz, H-5), 3.91 (1H, dd, *J* 4.8, 2.9 Hz, H-1), 3.66 (1H, d, *J* 13.3 Hz, CH_2Ph), 3.52 (1H, d, *J* 13.3 Hz, CH_2Ph), 2.86 (1H, dt, *J* 10.4, 6.4 Hz, H-2), 2.31 (3H, s, CH_3), 2.29-2.25 (1H, m, CH_2), 2.13 (3H, s, NCH_3), 2.09-1.97 (1H, m, CH_2), 1.86-1.67 (2H, m, CH_2); d_C (100 MHz, DMSO-d_6) 143.9, 139.0, 128.5, 127.9, 126.6, 116.0, 76.3, 66.1, 62.7, 59.3, 28.2, 26.2, 12.9; LC-MS (APCI): *m/z* = 286.16 $[M]^+$. HRMS (ESI): *m/z* calcd for $[C_{17}H_{23}N_3O]^+$: 286.1914; found: 286.1920.

(1*RS*,2*RS*,5*RS*)-2-(6-Amino-9*H*-purin-9-yl)-5-[benzyl(methyl)amino]cyclopentanol (**8d**)

This compound was isolated in 65% yield (1.10 g) as a brown solid; d_H (400 MHz, DMSO-d_6) 8.15 (1H, s, CH), 8.13 (1H, s, CH), 7.35-7.18 (5H, m, Ph), 7.04 (2H, s, NH_2), 4.75 (1H, td, *J* 8.3, 3.2 Hz, H-2), 4.70 (1H, s, OH), 4.30-4.22 (1H, m, H-1), 3.68 (1H, d, *J* 13.3 Hz, CH_2Ph), 3.56 (1H, d, *J* 13.3 Hz, CH_2Ph), 3.11 (1H, dt, *J* 12.0, 6.1 Hz, H-5), 2.37-2.26 (1H, m, H-3), 2.15 (3H, s, CH_3), 2.12-2.00 (2H, m, H-3 and H-4), 1.90-1.80 (1H, m, H-4); d_C (100 MHz, DMSO-d_6, HSQC-DEPT) 128.0, 127.5, 126.3 (Ph), 74.8 (C-1), 65.6 (C-5), 61.7 (C-2), 58.9 (CH_2Ph), 39.2 (CH_3), 27.2 (C-3), 26.0 (C-4); LC-MS (APCI): *m/z* = 339.27 $[M]^+$. HRMS (ESI): *m/z* calcd for $[C_{18}H_{22}N_6O]^+$: 339.1928; found: 339.1923.

(1*RS*,2*RS*,5*RS*)-2-(Dibenzylamino)-5-(2-methyl-1*H*-imidazol-1-yl)cyclopentanol (**9b**)

This compound was isolated in 64% yield (1.15 g) as a yellow oil, and regioisomer **10b** was obtained in 26% yield (0.47 g) as a white solid; *Compound **9b***: d_H (400 MHz, DMSO-d_6) 7.39-7.14 (10H, m, Ph), 6.90 (1H, d, *J* 1.1 Hz, CH), 6.69 (1H, s, CH), 4.99 (1H, s, OH), 4.29 (1H, td, *J* 8.4, 4.8 Hz, H-5), 3.98 (1H, d, *J* 5.1 Hz, H-1), 3.81 (2H, d, *J* 14.2 Hz, CH_2Ph), 3.75 (2H, d, *J* 14.2 Hz, CH_2Ph), 3.20-3.12 (1H, m, H-2), 2.30 (3H, s, CH_3), 2.16-2.05 (1H, m, CH_2), 1.94-1.75 (2H, m, CH_2), 1.66-1.53 (1H, m, CH_2); d_C (100 MHz, DMSO-d_6) 154.5, 144.2, 140.3, 128.4, 128.0, 126.6, 115.8, 77.0, 63.1, 61.6, 55.5, 28.0, 25.2, 13.1; LC-MS (APCI): *m/z* = 362.29 $[M]^+$. HRMS (ESI): *m/z* calcd for $[C_{23}H_{27}N_3O]^+$: 362.2227; found: 362.2227. *Compound **10b***: d_H (400 MHz, DMSO-d_6) 7.25-7.07 (10H, m, Ph), 6.76 (1H, s, CH), 6.74 (1H, s, CH), 4.98 (1H, d, *J* 4.0 Hz, OH), 4.20 (1H, dd, *J* 9.3, 7.8 Hz, H-2), 3.95-3.84 (1H, m, H-1), 3.61 (2H, d, *J* 14.3 Hz, CH_2Ph), 3.52 (2H, d, *J* 14.3 Hz, CH_2Ph), 3.38 (1H, dd, *J* 17.7, 8.6 Hz, H-3), 2.23 (3H, s, CH_3), 1.97-1.73 (3H, m, H-4 and H-5), 1.64-1.52 (1H, m, H-4); d_C (100 MHz, DMSO-d_6, HMBC) 127.5, 127.4, 126.0 (Ph), 125.8 (CH), 115.6 (CH), 74.4 (C-1), 64.1 (C-2), 62.4 (C-3), 53.2 (CH_2Ph), 30.0 (C-4), 20.0 (C-5), 12.8 (CH_3); LC-MS (APCI): *m/z* = 362.29 $[M]^+$. HRMS (ESI): *m/z* calcd for $[C_{23}H_{27}N_3O]^+$: 362.2227; found: 362.2222.

(1*RS*,2*RS*,5*RS*)-2-(6-Amino-9*H*-purin-9-yl)-5-(dibenzylamino)cyclopentanol (**9d**)

This compound was isolated in 46% yield (0.95 g) as a brown solid, and regioisomer **10d** was obtained in 23% yield (0.47 g) as a brown solid; *Compound **9d***: d_H (400 MHz, DMSO-d_6) 8.06 (2H, s, CH), 7.41-7.14 (10H, m, Ph), 7.02 (2H, s, NH_2), 5.03 (1H, d, *J* 6.9 Hz, OH), 4.66 (1H, dd, *J* 13.0, 8.1 Hz, H-2), 4.38 (1H, t, *J* 5.5 Hz, H-1), 3.79 (4H, dd, *J* 29.2, 14.2 Hz, CH_2Ph), 3.43 (1H, dt, *J* 23.4, 8.3 Hz, H-5), 2.21-2.10 (1H, m, H-3), 2.02-1.91 (2H, m, H-3 and H-4), 1.89-1.80 (1H, m, H-4); d_C (100 MHz, DMSO-d_6, HMBC) 151.8 (CH), 139.5 (CH), 128.2, 127.9, 126.3 (Ph), 75.1 (C-2), 62.6 (C-1), 61.1 (CH_2Ph), 55.2 (C-5), 26.9 (C-4), 25.1 (C-3); LC-MS (APCI): *m/z* = 415.28 $[M]^+$. HRMS (ESI): *m/z* calcd for $[C_{24}H_{26}N_6O]^+$: 415.2241; found: 415.2248. *Compound **10d***: d_H (400 MHz, DMSO-d_6) 8.02 (1H, s, CH), 7.92 (1H, s, CH), 7.10-6.92 (10H, m, Ph), 7.00 (2H, buried s, NH_2), 5.02 (1H, d, *J* 4.6 Hz, OH), 4.61 (1H, dd, *J* 9.8, 7.9 Hz, H-2), 4.53 (1H, dd, *J* 11.5, 7.1 Hz, H-1), 3.71 (1H, dd, *J* 18.3, 8.7 Hz, H-3), 3.62 (2H, d, *J* 13.9 Hz, CH_2Ph), 3.44 (2H, d, *J* 13.9 Hz, CH_2Ph), 2.05-1.96 (1H, m, H-5), 1.94-1.74 (2H, m, H-4), 1.69-1.56 (1H, m, H-5); d_C (100 MHz, DMSO-d_6,

HMBC) 155.8 (CH), 139.3 (CH), 128.1, 127.7, 126.4 (Ph), 71.1 (C-2), 61.0 (C-1), 52.9 (C-3), 52.1 (CH_2Ph), 30.1 (C-4), 20.1 (C-5); LC-MS (APCI): *m/z* = 415.34 $[M]^+$. HRMS (ESI): *m/z* calcd for $[C_{24}H_{26}N_6O]^+$: 415.2241; found: 415.2229.

(1*SR*,2*SR*,5*RS*)-2-(6-Amino-9*H*-purin-9-yl)-5-[benzyl(methyl)amino]cyclopentanol (**13a**)

This compound was isolated in 68% yield (1.15 g) as a yellow solid; d_H (400 MHz, C_5D_5N) 8.60 (1H, s, CH), 8.32 (1H, s, CH), 7.83 (2H, s, NH_2), 7.52-7.22 (5H, m, Ph), 5.08 (1H, t, *J* 8.1 Hz, H-1), 4.85 (1H, dd, *J* 18.0, 8.6 Hz, H-2), 4.63 (1H, s, OH), 3.87 (2H, q, *J* 13.6 Hz, CH_2Ph), 3.29 (1H, dd, *J* 16.3, 7.9 Hz, H-5), 2.50-2.40 (1H, m, H-3), 2.37 (3H, s, CH_3), 2.27-2.13 (1H, m, H-3), 2.10-1.89 (2H, m, H-4); d_C (100 MHz, C_5D_5N, HMBC) 151.0 (CH), 140.7 (CH), 128.6, 127.2, 123.6 (Ph), 77.5 (C-2), 69.2 (CH_3), 63.2 (C-1), 60.0 (C-5), 38.8 (CH_2Ph), 26.7 (C-4), 23.5 (C-3); LC-MS (APCI): *m/z* = 338.71 $[M]^+$. HRMS (ESI): *m/z* calcd for $[C_{18}H_{22}N_6O]^+$: 339.1928; found: 339.1930.

(1*SR*,2*SR*,5*RS*)-2-(6-Amino-9*H*-purin-9-yl)-5-(dibenzylamino)cyclopentanol (**13b**)

This compound was isolated in 55% yield (1.14 g) as a brown solid; d_H (400 MHz, DMSO-d_6) 8.15 (1H, s, CH), 8.10 (1H, s, CH), 7.44-7.14 (10H, m, Ph), 7.00 (2H, s, NH_2), 5.21 (1H, d, *J* 5.4 Hz, OH), 4.68 (1H, dd, *J* 13.8, 8.4 Hz, H-1), 4.43 (1H, dd, *J* 18.2, 9.0 Hz, H-2), 3.84 (2H, d, *J* 14.1 Hz, CH_2Ph), 3.67 (2H, d, *J* 14.2 Hz, CH_2Ph), 3.17-3.10 (1H, m, H-5), 2.19-1.98 (2H, m, H-3), 1.95-1.74 (2H, m, H-4); d_C (100 MHz, DMSO-d_6, HSQC-DEPT) 151.3 (CH), 139.5 (CH), 128.0, 127.6, 126.1 (Ph), 74.9 (C-1), 63.3 (C-5), 60.3 (C-2), 53.6 (CH_2Ph), 25.6 (C-3), 20.7 (C-4); LC-MS (APCI): *m/z* = 415.27 $[M]^+$. HRMS (ESI): *m/z* calcd for $[C_{24}H_{26}N_6O]^+$: 415.2241; found: 415.2229.

References

1. Bond, C. W.; Cresswell, A. J.; Davies, S. G.; Fletcher, A. M.; Kurosawa, W.; Lee, J. A.; Roberts, P. M.; Russell, A. J.; Smith, A. D.; Thomson, J. E. *J. Org. Chem*. **2009**, *74*, 6735–6748.
2. Brennan, M.; Claridge, T.; Compton, R.; Davies, S. *J. Org. Chem.* **2012**, *77*, 7241-7261.
