# Supplementary material for: Regio- and stereoselective synthesis of new diaminocyclopentanols
Source: Beilstein J Org Chem. 2014 Oct 28;10:2513–20. doi: 10.3762/bjoc.10.262 (PMC4222406; doi:10.3762/bjoc.10.262)
Supplement: File 2 — Copies of 1H and 13C NMR spectra. [file Beilstein_J_Org_Chem-10-2513-s002.pdf]

**Supporting Information**

**for**

# Regio- and stereoselective synthesis of new diaminocyclopentanols

Evgeni A. Larin*[1], Valeri S. Kochubei[1] and Yuri M. Atroshchenko[2]

Address: [1]Organic Synthesis Department, Asinex Corporation, 101 North Chestnut, Winston-Salem 27101, NC, USA and [2]Organic Chemistry and Biochemistry Department, Tolstoi State Pedagogical University, 126 Lenin, Tula 300026, Russian Federation

Email: Evgeni Anatolyevich Larin* – elarin@asinex.com

* Corresponding author

## Copies of $^{1}$H and $^{13}$C NMR spectra

**Table of contents**

$^{1}$H NMR spectrum of **3a** S-3
$^{1}$H NMR spectrum of **3b** S-4
$^{1}$H NMR spectrum of **3c** S-5
$^{1}$H NMR spectrum of **3d** S-6
$^{1}$H NMR spectrum of **6a** S-7
$^{1}$H NMR spectrum of **6b** S-8
$^{1}$H NMR spectrum of **8a** S-9
$^{1}$H NMR spectrum of **8b** S-10
$^{13}$C NMR spectrum of **8b** S-11
$^{1}$H NMR spectrum of **8c** S-12

| | |
|---|---|
| $^{13}C$ NMR spectrum of **8c** | S-13 |
| $^{1}H$ NMR spectrum of **8d** | S-14 |
| $^{1}H$ NMR spectrum of **9a** | S-15 |
| $^{1}H$ NMR spectrum of **10a** | S-16 |
| $^{1}H$ NMR spectrum of **9b** | S-17 |
| $^{13}C$ NMR spectrum of **9b** | S-18 |
| $^{1}H$ NMR spectrum of **10b** | S-19 |
| $^{1}H$ NMR spectrum of **9c** | S-20 |
| $^{13}C$ NMR spectrum of **9c** | S-21 |
| $^{1}H$ NMR spectrum of **10c** | S-22 |
| $^{1}H$ NMR spectrum of **9d** | S-23 |
| $^{1}H$ NMR spectrum of **10d** | S-24 |
| $^{1}H$ NMR spectrum of the mixture of **11** and **12** | S-25 |
| $^{1}H$ NMR spectrum of **13a** | S-26 |
| $^{1}H$ NMR spectrum of **13b** | S-27 |
| $^{1}H$ NMR spectrum of **14a** | S-28 |
| $^{1}H$ NMR spectrum of **14b** | S-29 |
| $^{1}H$ NMR spectrum of **15** | S-30 |

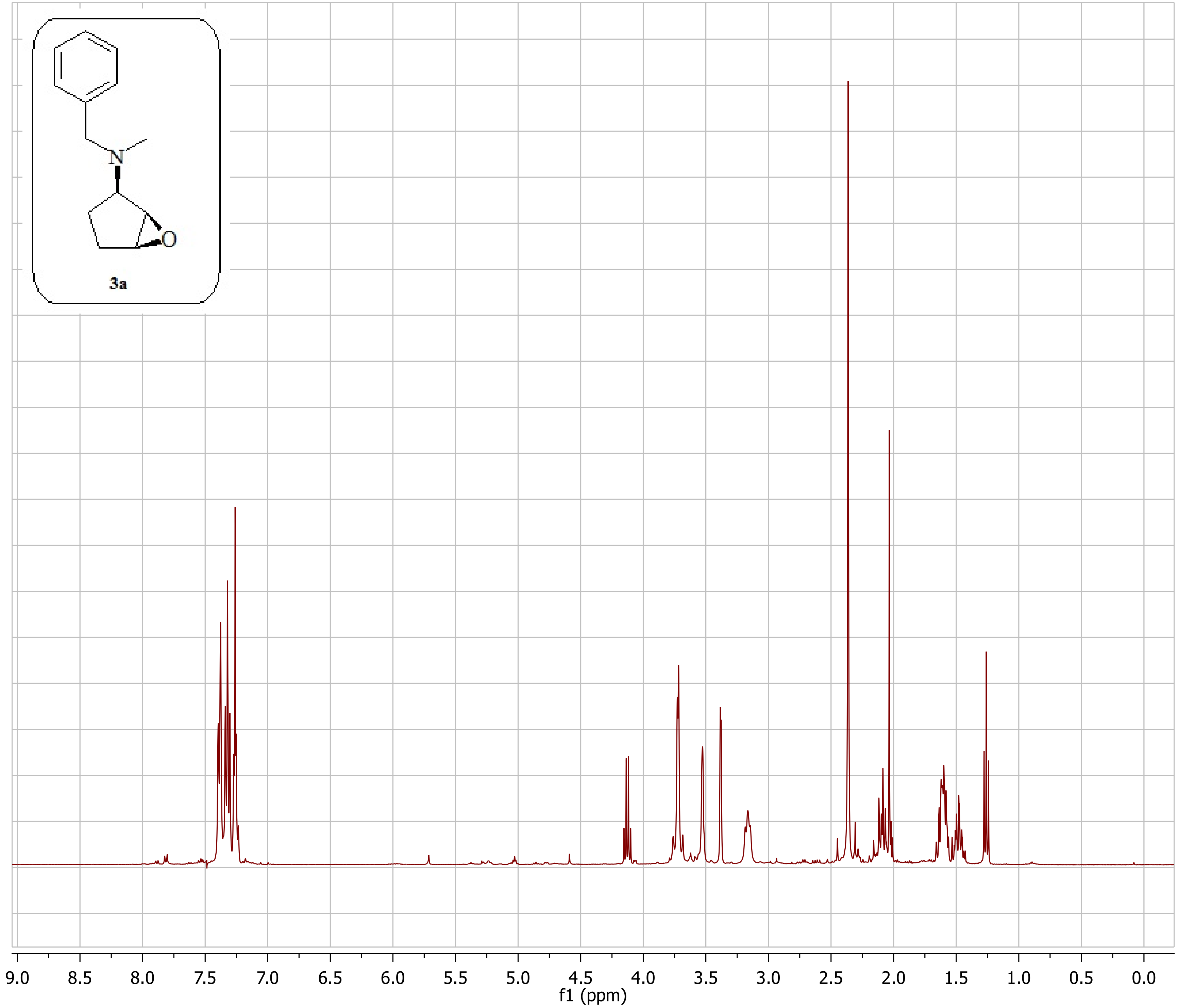


| | Параметр | Значение |
|---|---|---|
| 1 | Origin | Varian |
| 2 | Spectrometer | mercury |
| 3 | Author | vnmr1 |
| 4 | Solvent | CDCl3 |
| 5 | Temperature | 50.0 |
| 6 | Pulse Sequence | s222 |
| 7 | Number of Scans | 10 |
| 8 | Receiver Gain | 16 |
| 9 | Relaxation Delay | 3.0000 |
| 10 | Pulse Width | 0.0000 |
| 11 | Acquisition Time | 1.4000 |
| 12 | Gradient Duration | 0.0020 |
| 13 | Spectrometer Frequency | 400.45 |
| 14 | Spectral Width | 6802.7 |
| 15 | Lowest Frequency | -403.2 |
| 16 | Nucleus | 1H |
| 17 | Acquired Size | 9524 |
| 18 | Spectral Size | 32768 |

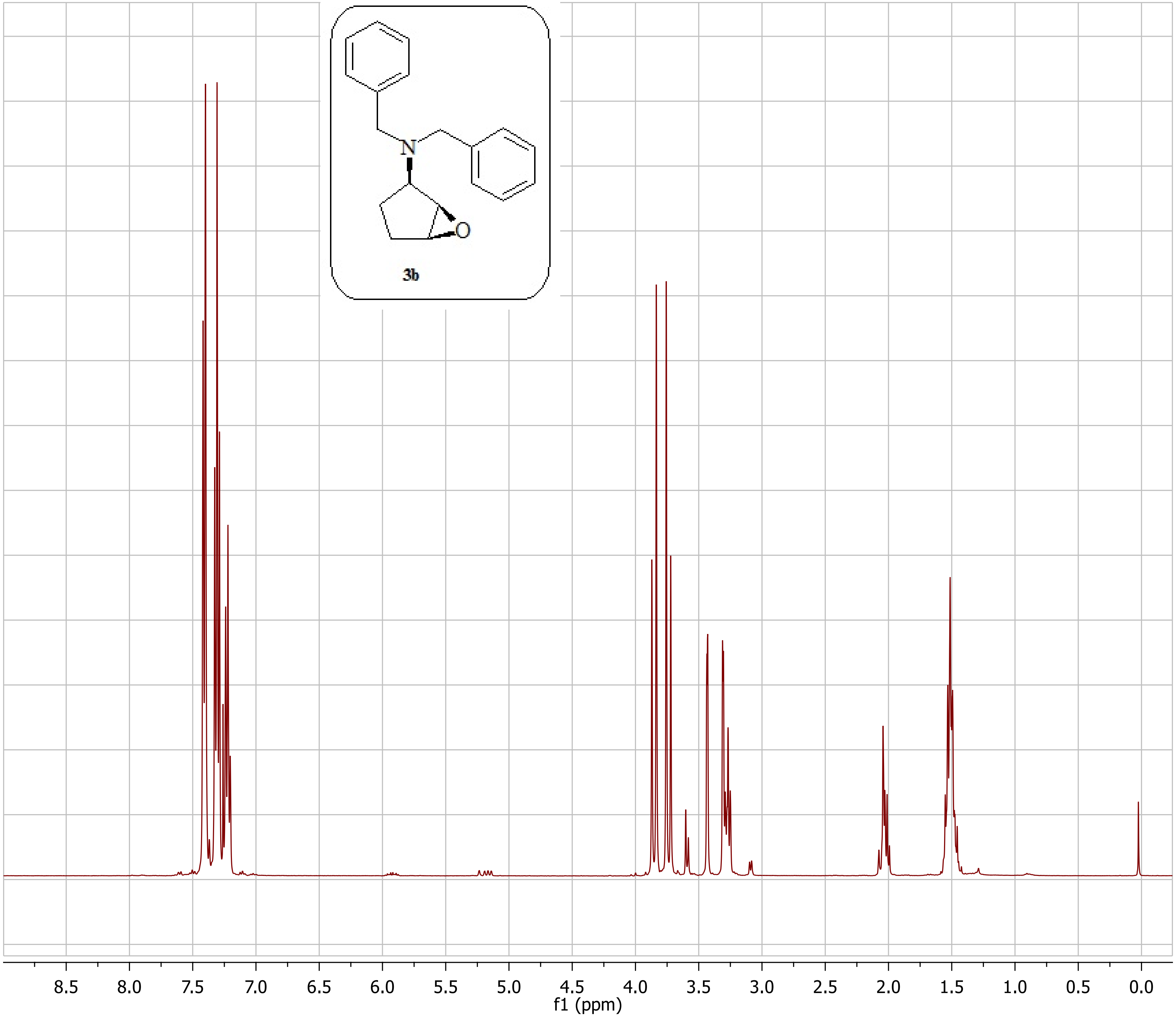


3b

| | Параметр | Значение |
|---|---|---|
| 1 | Origin | Varian |
| 2 | Spectrometer | mercury |
| 3 | Author | vnmr1 |
| 4 | Solvent | CDCl3 |
| 5 | Temperature | 50.0 |
| 6 | Pulse Sequence | s222 |
| 7 | Number of Scans | 4 |
| 8 | Receiver Gain | 16 |
| 9 | Relaxation Delay | 3.0000 |
| 10 | Pulse Width | 0.0000 |
| 11 | Acquisition Time | 1.4000 |
| 12 | Gradient Duration | 0.0020 |
| 13 | Spectrometer Frequency | 400.45 |
| 14 | Spectral Width | 6802.7 |
| 15 | Lowest Frequency | -403.2 |
| 16 | Nucleus | 1H |
| 17 | Acquired Size | 9524 |
| 18 | Spectral Size | 32768 |

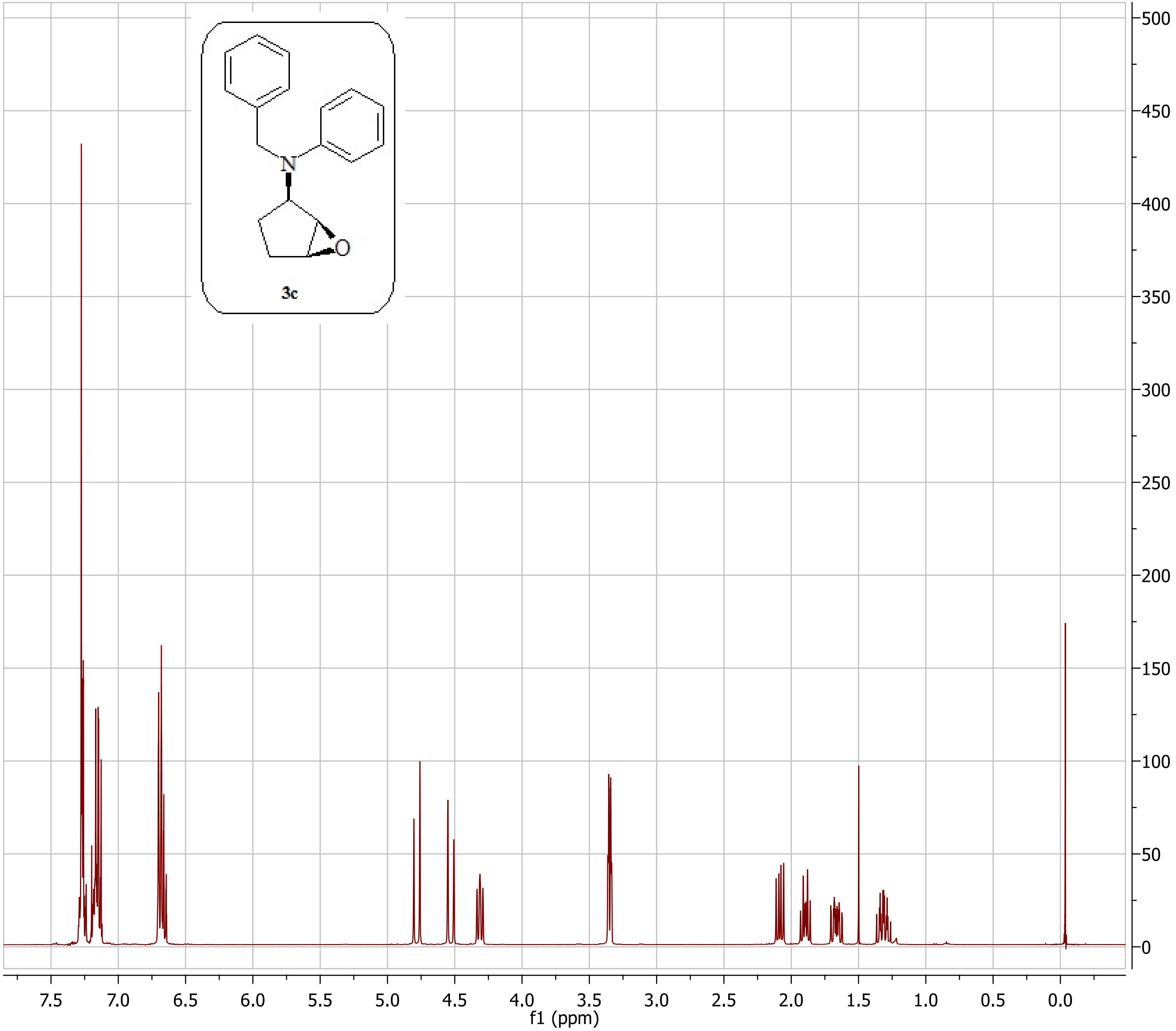


| | Параметр | Значение |
|---|---|---|
| 1 | Origin | Varian |
| 2 | Spectrometer | vnmrs |
| 3 | Author | chemp |
| 4 | Solvent | cdcl3 |
| 5 | Temperature | 34.0 |
| 6 | Pulse Sequence | s2pul |
| 7 | Number of Scans | 16 |
| 8 | Receiver Gain | 30 |
| 9 | Relaxation Delay | 1.0000 |
| 10 | Pulse Width | 0.0000 |
| 11 | Acquisition Time | 2.5559 |
| 12 | Gradient Duration | 0.0010 |
| 13 | Spectrometer Frequency | 399.70 |
| 14 | Spectral Width | 6410.3 |
| 15 | Lowest Frequency | -825.5 |
| 16 | Nucleus | 1H |
| 17 | Acquired Size | 16384 |
| 18 | Spectral Size | 32768 |

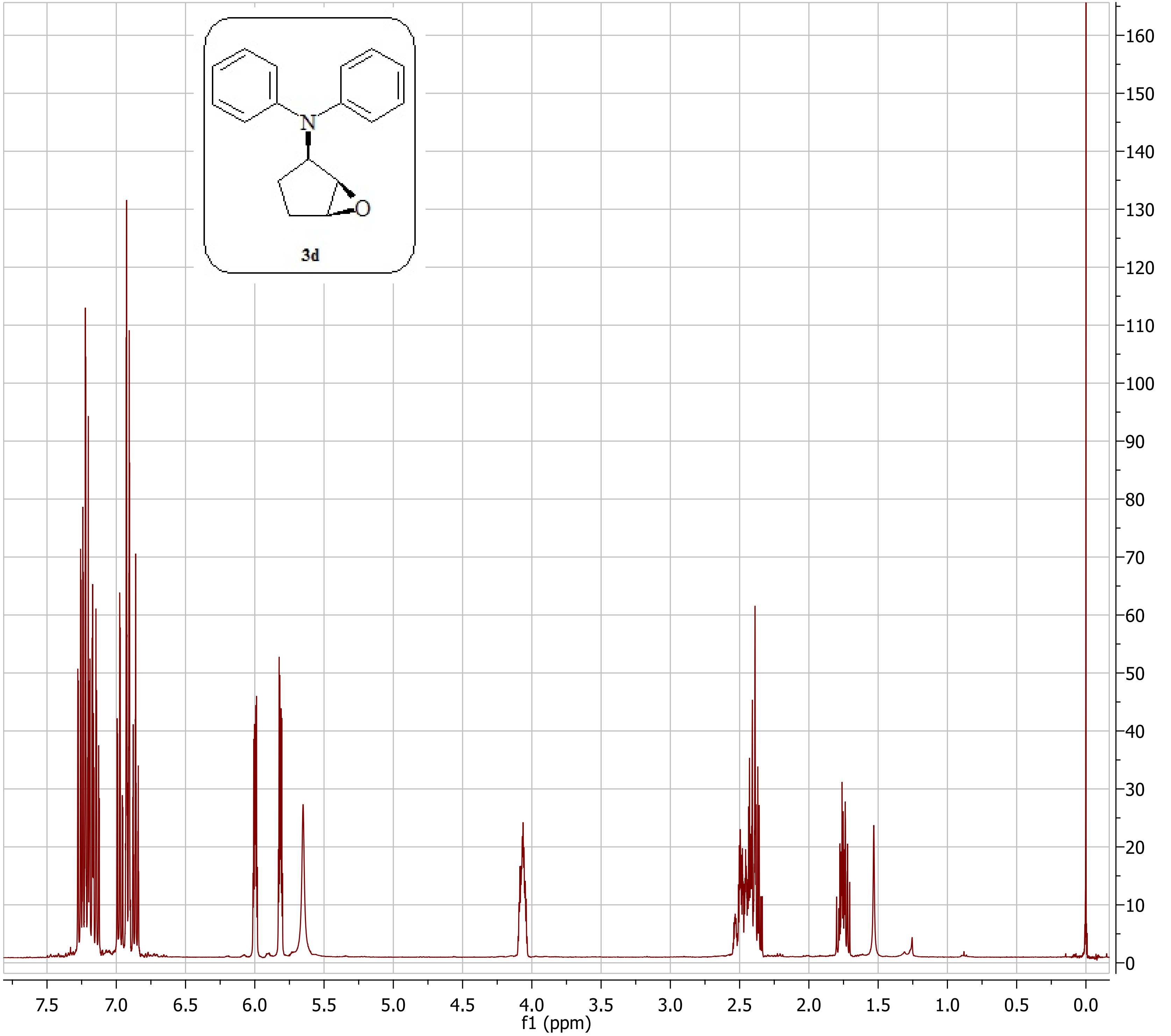


| | Параметр | Значение |
|---|---|---|
| 1 | Origin | Varian |
| 2 | Spectrometer | vnmrs |
| 3 | Author | chemp |
| 4 | Solvent | cdcl3 |
| 5 | Temperature | 34.0 |
| 6 | Pulse Sequence | s2pul |
| 7 | Number of Scans | 16 |
| 8 | Receiver Gain | 30 |
| 9 | Relaxation Delay | 1.0000 |
| 10 | Pulse Width | 0.0000 |
| 11 | Acquisition Time | 2.5559 |
| 12 | Gradient Duration | 0.0010 |
| 13 | Spectrometer Frequency | 399.70 |
| 14 | Spectral Width | 6410.3 |
| 15 | Lowest Frequency | -808.3 |
| 16 | Nucleus | 1H |
| 17 | Acquired Size | 16384 |
| 18 | Spectral Size | 32768 |

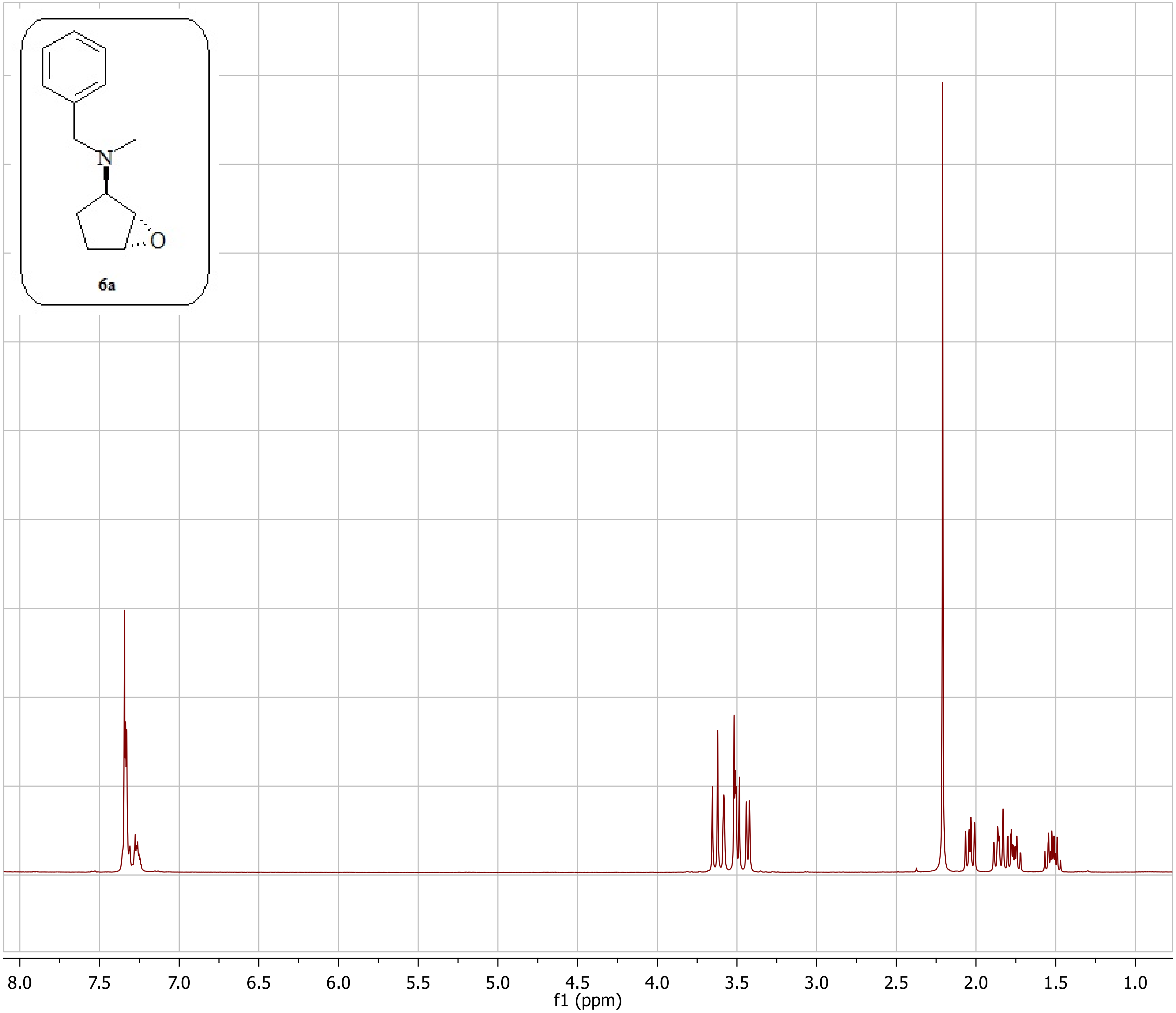


| | Параметр | Значение |
|---|---|---|
| 1 | Origin | Varian |
| 2 | Spectrometer | mercury |
| 3 | Author | vnmr1 |
| 4 | Solvent | CDCl3 |
| 5 | Temperature | 63.0 |
| 6 | Pulse Sequence | s2pul |
| 7 | Number of Scans | 16 |
| 8 | Receiver Gain | 4 |
| 9 | Relaxation Delay | 3.0000 |
| 10 | Pulse Width | 0.0000 |
| 11 | Acquisition Time | 1.4022 |
| 12 | Gradient Duration | 0.0020 |
| 13 | Spectrometer Frequency | 400.45 |
| 14 | Spectral Width | 2936.9 |
| 15 | Lowest Frequency | 307.4 |
| 16 | Nucleus | 1H |
| 17 | Acquired Size | 4118 |
| 18 | Spectral Size | 16384 |

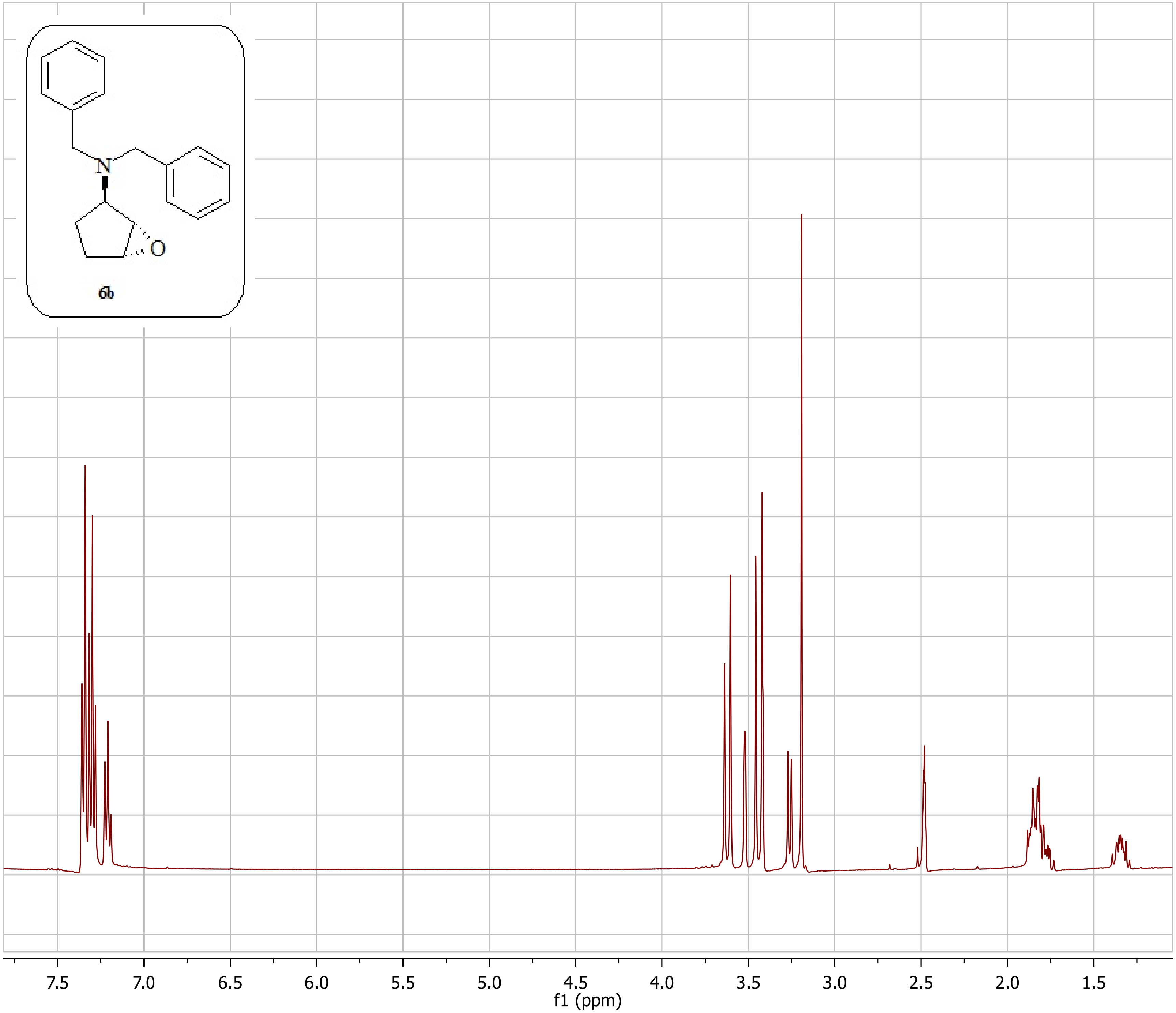


| | Параметр | Значение |
|---|---|---|
| 1 | Origin | Varian |
| 2 | Spectrometer | mercury |
| 3 | Author | vnmr1 |
| 4 | Solvent | DMSO |
| 5 | Temperature | 50.0 |
| 6 | Pulse Sequence | s2pul |
| 7 | Number of Scans | 16 |
| 8 | Receiver Gain | 8 |
| 9 | Relaxation Delay | 3.0000 |
| 10 | Pulse Width | 0.0000 |
| 11 | Acquisition Time | 2.5000 |
| 12 | Gradient Duration | 0.0020 |
| 13 | Spectrometer Frequency | 400.45 |
| 14 | Spectral Width | 2710.0 |
| 15 | Lowest Frequency | 418.5 |
| 16 | Nucleus | 1H |
| 17 | Acquired Size | 6775 |
| 18 | Spectral Size | 16384 |

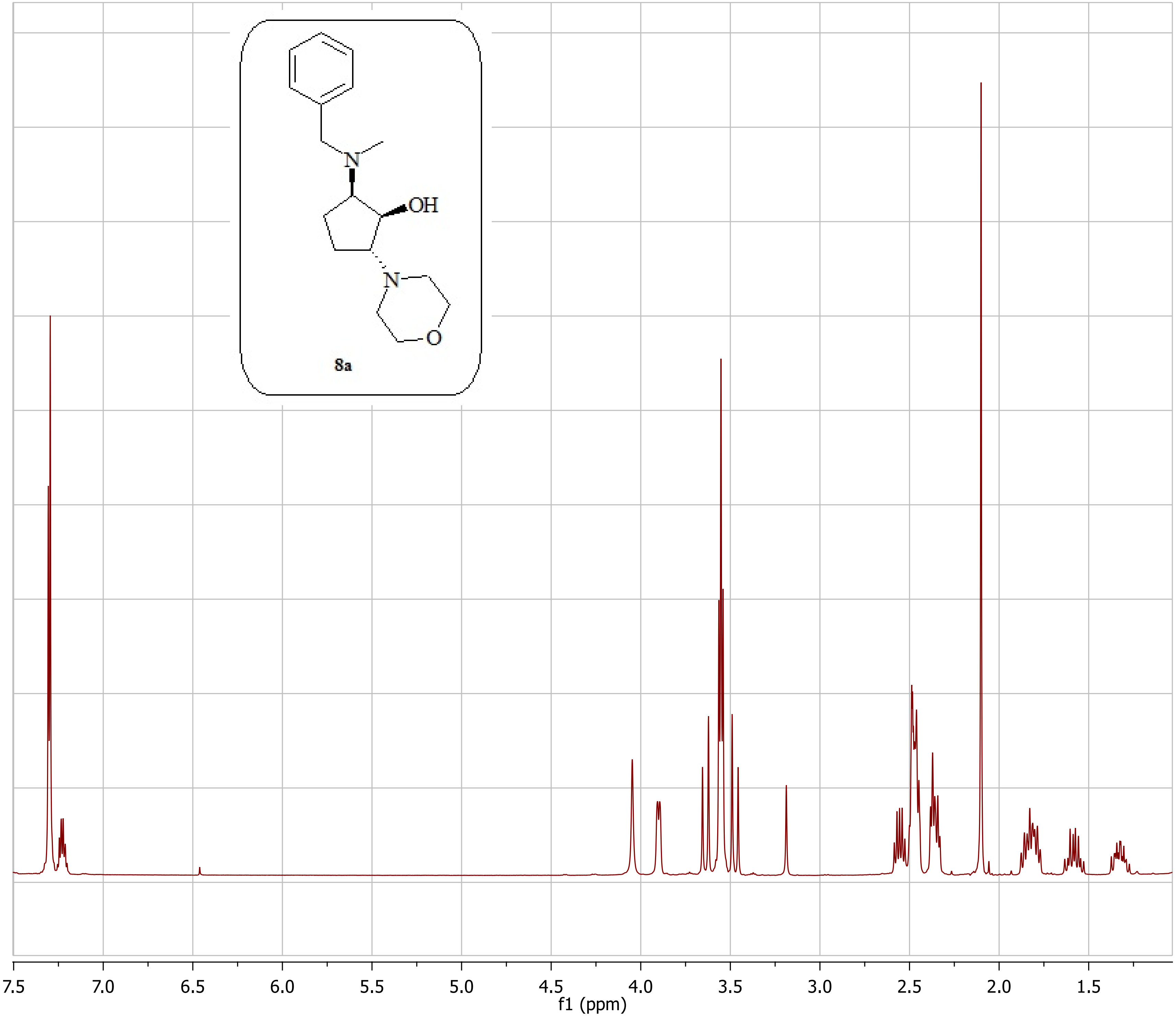


| | Параметр | Значение |
|---|---|---|
| 1 | Origin | Varian |
| 2 | Spectrometer | mercury |
| 3 | Author | vnmr1 |
| 4 | Solvent | DMSO |
| 5 | Temperature | 50.0 |
| 6 | Pulse Sequence | s2pul |
| 7 | Number of Scans | 4 |
| 8 | Receiver Gain | 8 |
| 9 | Relaxation Delay | 3.0000 |
| 10 | Pulse Width | 0.0000 |
| 11 | Acquisition Time | 1.4012 |
| 12 | Gradient Duration | 0.0020 |
| 13 | Spectrometer Frequency | 400.45 |
| 14 | Spectral Width | 2591.3 |
| 15 | Lowest Frequency | 413.7 |
| 16 | Nucleus | 1H |
| 17 | Acquired Size | 3631 |
| 18 | Spectral Size | 8192 |

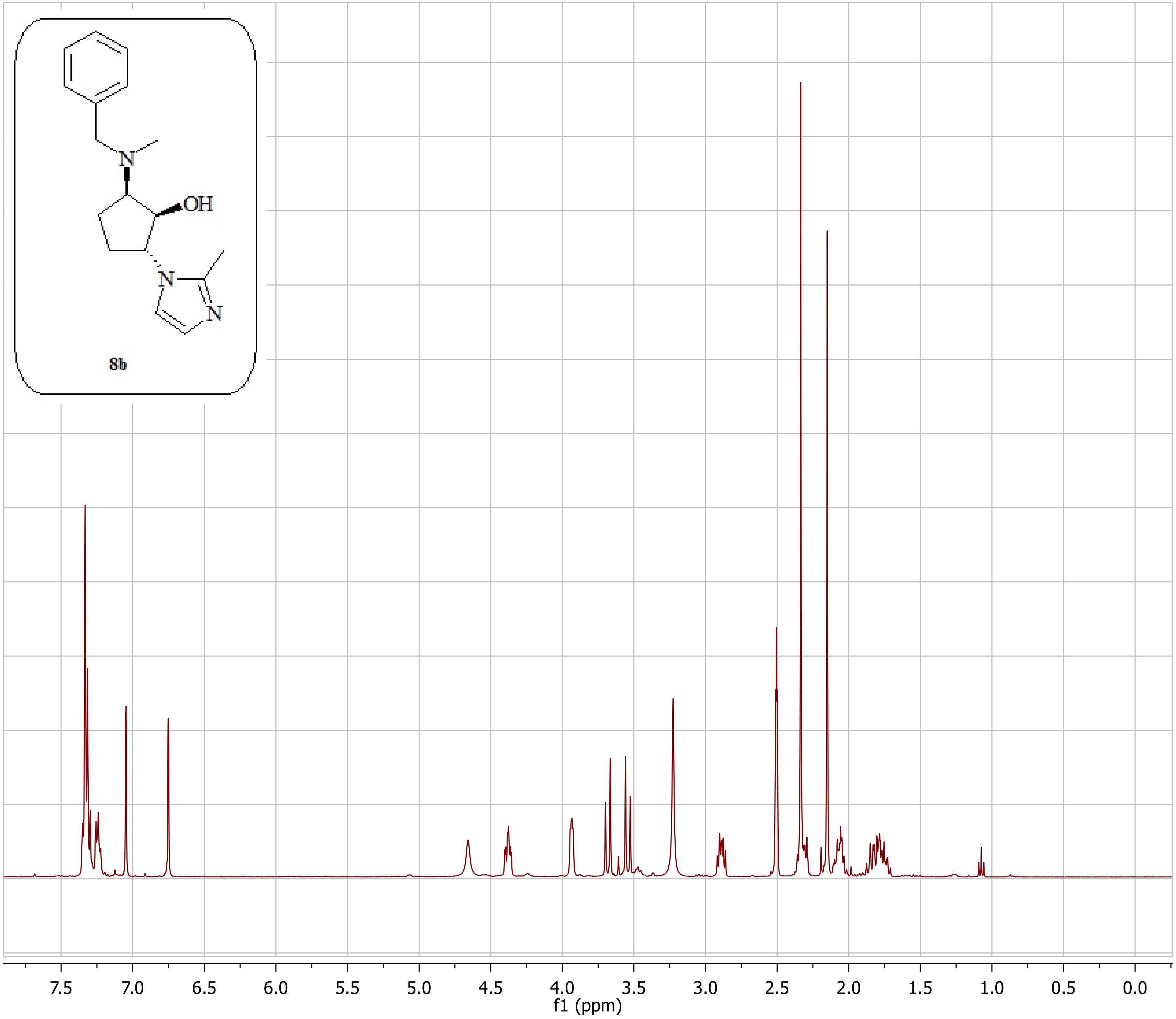


| | Параметр | Значение |
|---|---|---|
| 1 | Origin | Varian |
| 2 | Spectrometer | mercury |
| 3 | Author | vnmr1 |
| 4 | Solvent | DMSO |
| 5 | Temperature | 50.0 |
| 6 | Pulse Sequence | s222 |
| 7 | Number of Scans | 4 |
| 8 | Receiver Gain | 20 |
| 9 | Relaxation Delay | 3.0000 |
| 10 | Pulse Width | 0.0000 |
| 11 | Acquisition Time | 1.4000 |
| 12 | Gradient Duration | 0.0020 |
| 13 | Spectrometer Frequency | 400.46 |
| 14 | Spectral Width | 6535.9 |
| 15 | Lowest Frequency | -375.7 |
| 16 | Nucleus | 1H |
| 17 | Acquired Size | 9150 |
| 18 | Spectral Size | 32768 |

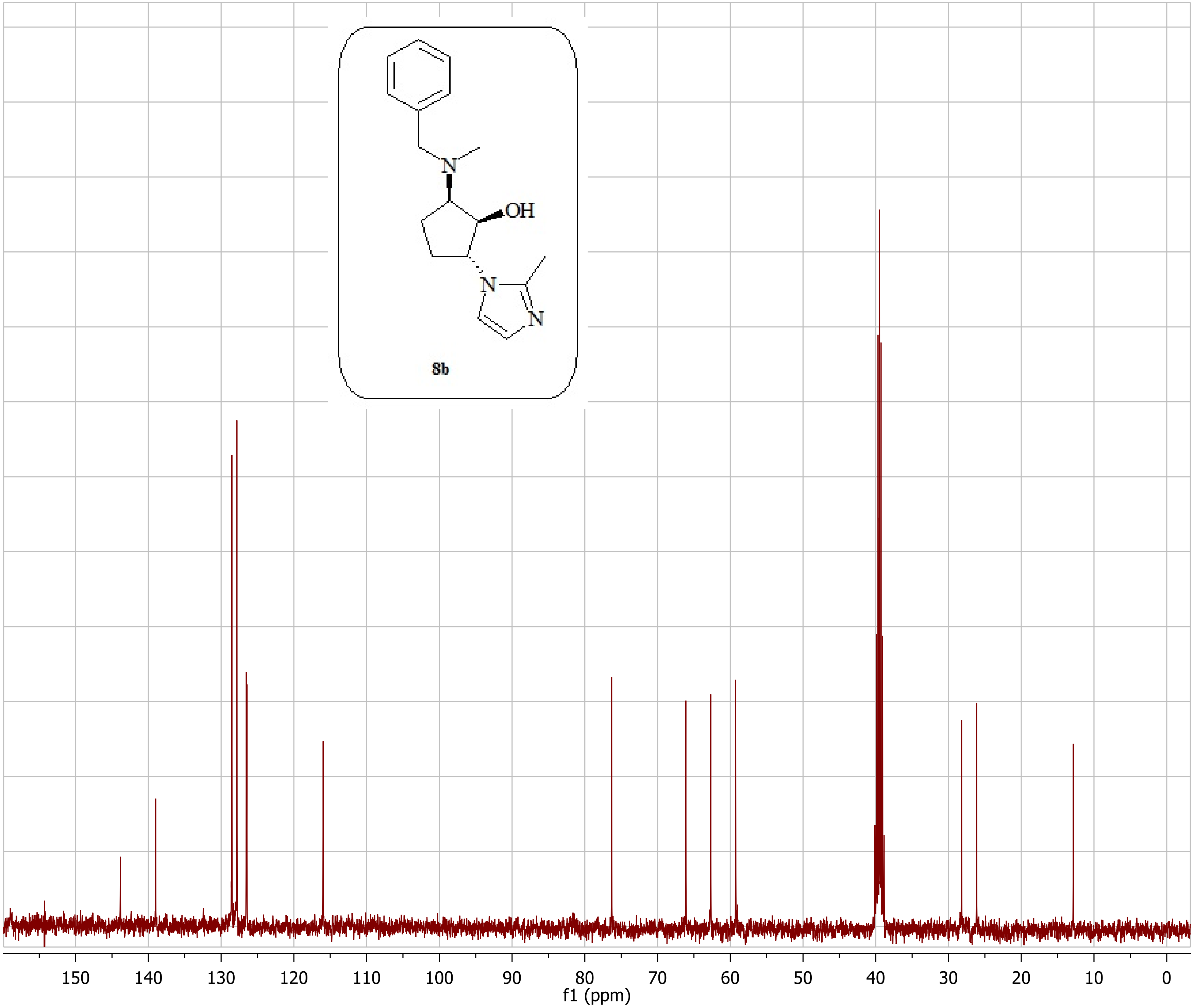


| | Параметр | Значение |
|---|---|---|
| 1 | Origin | Varian |
| 2 | Spectrometer | mercury |
| 3 | Author | vnmr1 |
| 4 | Solvent | dmso |
| 5 | Temperature | 50.0 |
| 6 | Pulse Sequence | s2pul |
| 7 | Number of Scans | 256 |
| 8 | Receiver Gain | 38 |
| 9 | Relaxation Delay | 3.0000 |
| 10 | Pulse Width | 0.0000 |
| 11 | Acquisition Time | 2.4448 |
| 12 | Gradient Duration | 0.0020 |
| 13 | Spectrometer Frequency | 100.71 |
| 14 | Spectral Width | 26178.0 |
| 15 | Lowest Frequency | -1087.7 |
| 16 | Nucleus | 13C |
| 17 | Acquired Size | 64000 |
| 18 | Spectral Size | 131072 |

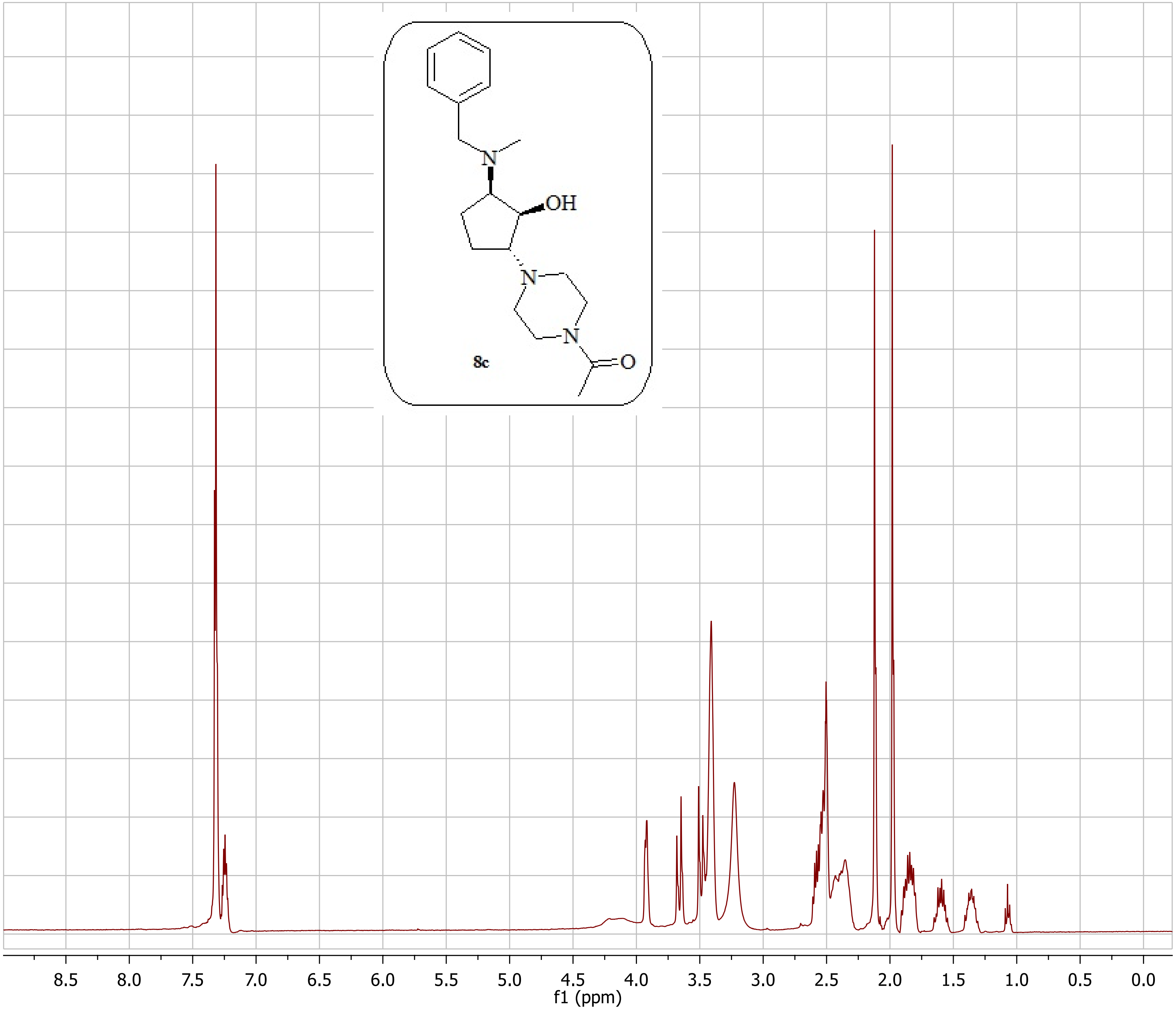


| | Параметр | Значение |
|---|---|---|
| 1 | Origin | Varian |
| 2 | Spectrometer | mercury |
| 3 | Author | vnmr1 |
| 4 | Solvent | DMSO |
| 5 | Temperature | 50.0 |
| 6 | Pulse Sequence | s222 |
| 7 | Number of Scans | 1 |
| 8 | Receiver Gain | 4 |
| 9 | Relaxation Delay | 3.0000 |
| 10 | Pulse Width | 0.0000 |
| 11 | Acquisition Time | 1.4000 |
| 12 | Gradient Duration | 0.0020 |
| 13 | Spectrometer Frequency | 400.46 |
| 14 | Spectral Width | 6535.9 |
| 15 | Lowest Frequency | -376.9 |
| 16 | Nucleus | 1H |
| 17 | Acquired Size | 9150 |
| 18 | Spectral Size | 32768 |

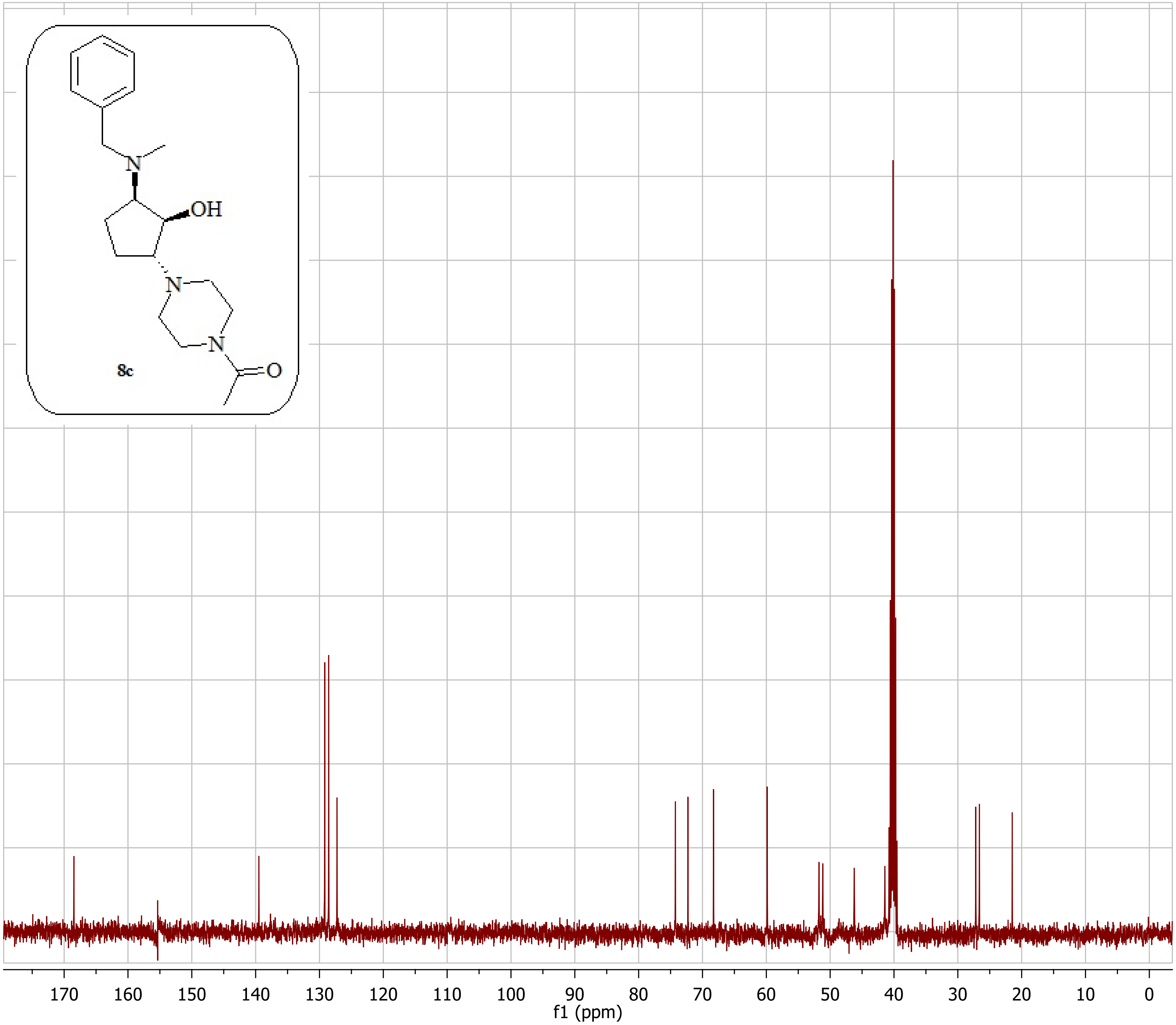


| | Параметр | Значение |
|---|---|---|
| 1 | Origin | Varian |
| 2 | Spectrometer | mercury |
| 3 | Author | vnmr1 |
| 4 | Solvent | dmso |
| 5 | Temperature | 50.0 |
| 6 | Pulse Sequence | s2pul |
| 7 | Number of Scans | 256 |
| 8 | Receiver Gain | 38 |
| 9 | Relaxation Delay | 3.0000 |
| 10 | Pulse Width | 0.0000 |
| 11 | Acquisition Time | 2.4448 |
| 12 | Gradient Duration | 0.0020 |
| 13 | Spectrometer Frequency | 100.71 |
| 14 | Spectral Width | 26178.0 |
| 15 | Lowest Frequency | -1020.9 |
| 16 | Nucleus | 13C |
| 17 | Acquired Size | 64000 |
| 18 | Spectral Size | 131072 |

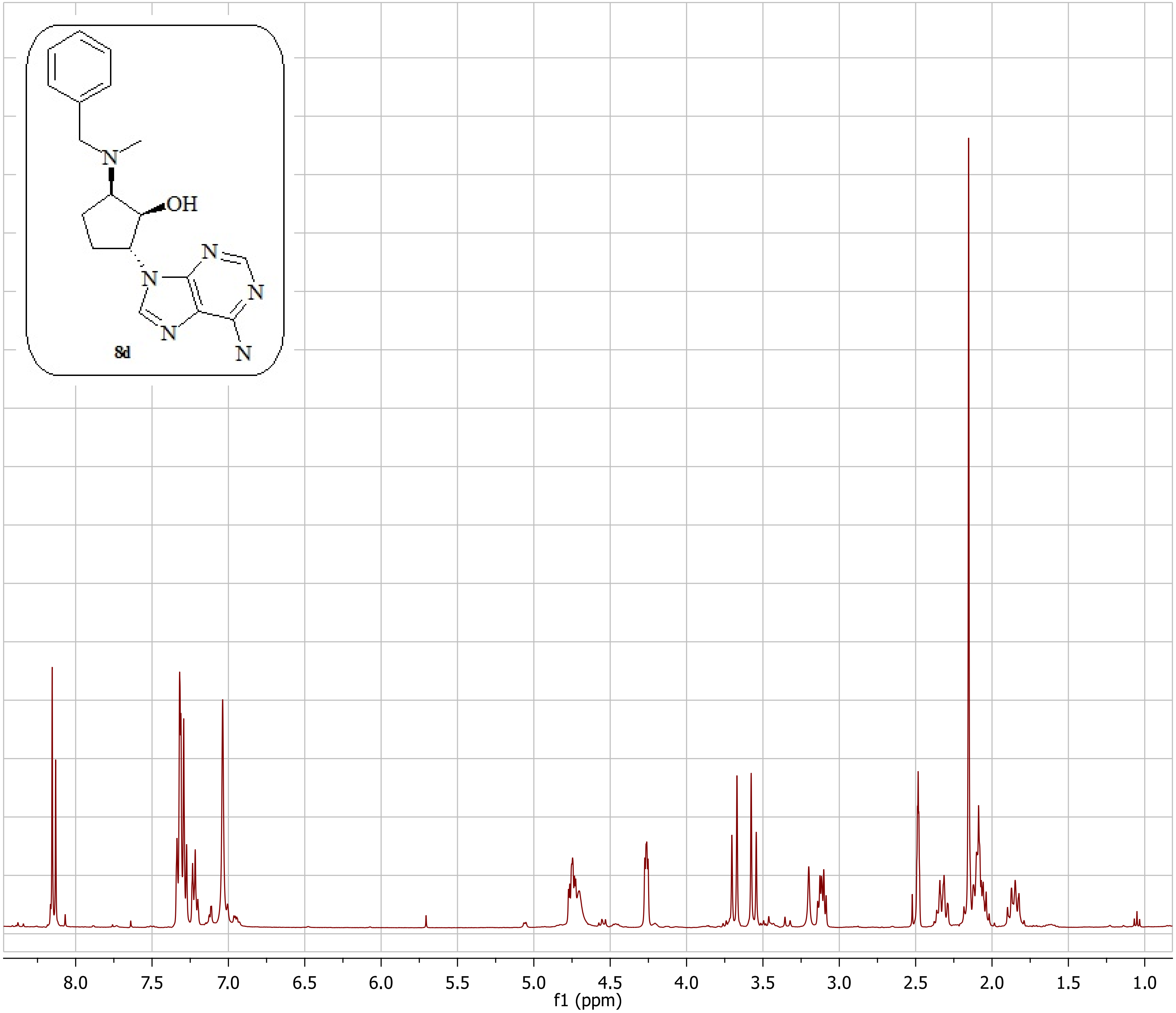


| | Параметр | Значение |
|---|---|---|
| 1 | Origin | Varian |
| 2 | Spectrometer | mercury |
| 3 | Author | vnmr1 |
| 4 | Solvent | DMSO |
| 5 | Temperature | 50.0 |
| 6 | Pulse Sequence | s2pul |
| 7 | Number of Scans | 4 |
| 8 | Receiver Gain | 8 |
| 9 | Relaxation Delay | 3.0000 |
| 10 | Pulse Width | 0.0000 |
| 11 | Acquisition Time | 1.3982 |
| 12 | Gradient Duration | 0.0020 |
| 13 | Spectrometer Frequency | 400.45 |
| 14 | Spectral Width | 3064.7 |
| 15 | Lowest Frequency | 327.9 |
| 16 | Nucleus | 1H |
| 17 | Acquired Size | 4285 |
| 18 | Spectral Size | 16384 |

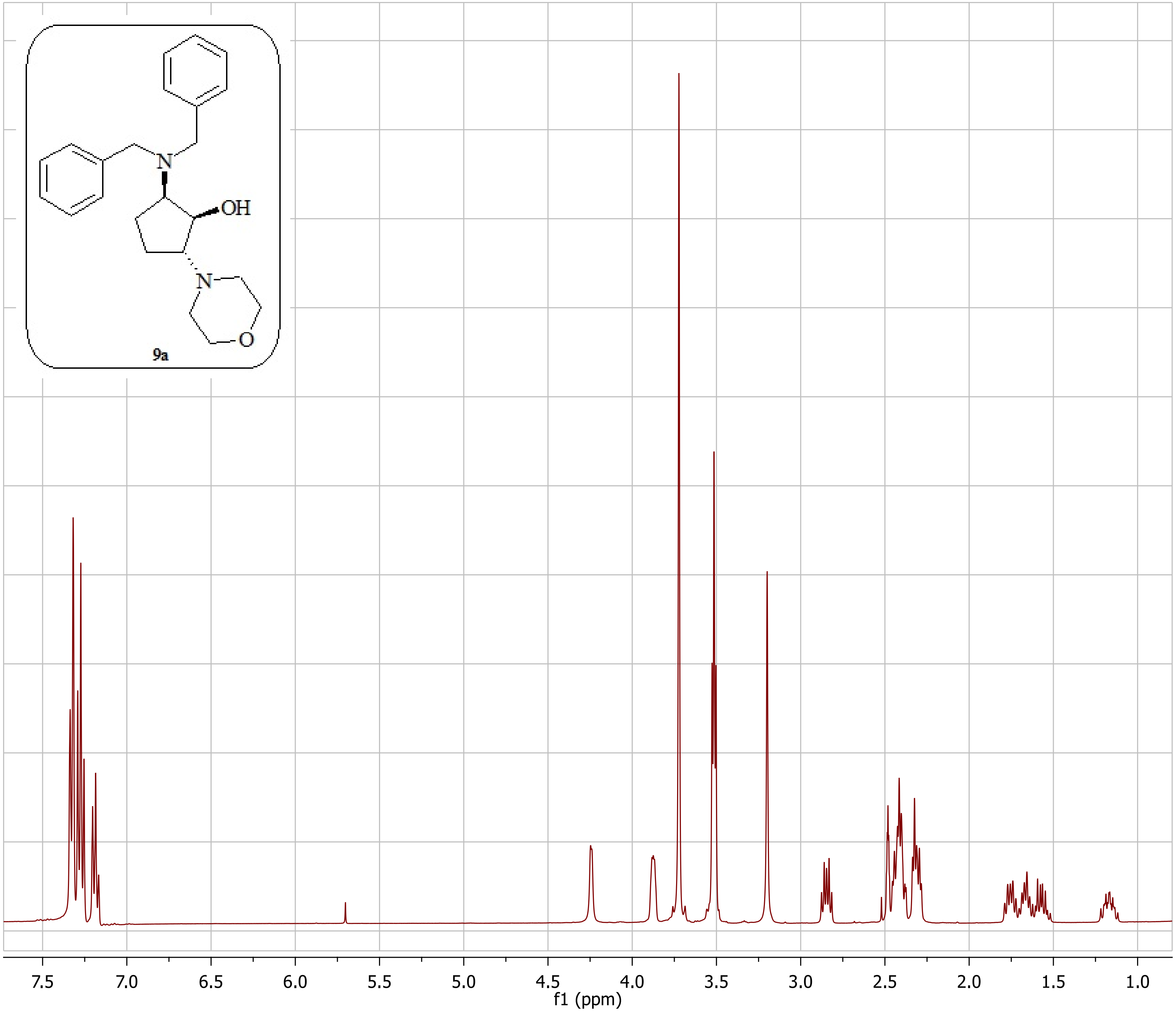


| | Параметр | Значение |
|---|---|---|
| 1 | Origin | Varian |
| 2 | Spectrometer | mercury |
| 3 | Author | vnmr1 |
| 4 | Solvent | DMSO |
| 5 | Temperature | 50.0 |
| 6 | Pulse Sequence | s2pul |
| 7 | Number of Scans | 16 |
| 8 | Receiver Gain | 8 |
| 9 | Relaxation Delay | 3.0000 |
| 10 | Pulse Width | 0.0000 |
| 11 | Acquisition Time | 2.4998 |
| 12 | Gradient Duration | 0.0020 |
| 13 | Spectrometer Frequency | 400.45 |
| 14 | Spectral Width | 2777.8 |
| 15 | Lowest Frequency | 318.3 |
| 16 | Nucleus | 1H |
| 17 | Acquired Size | 6944 |
| 18 | Spectral Size | 16384 |

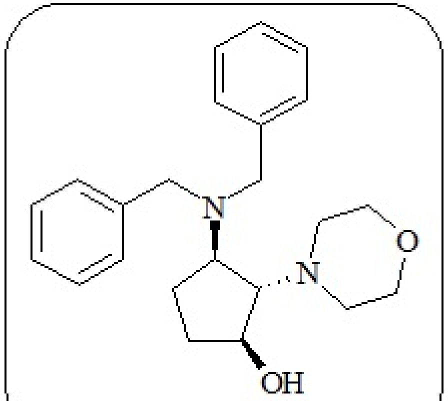


10a

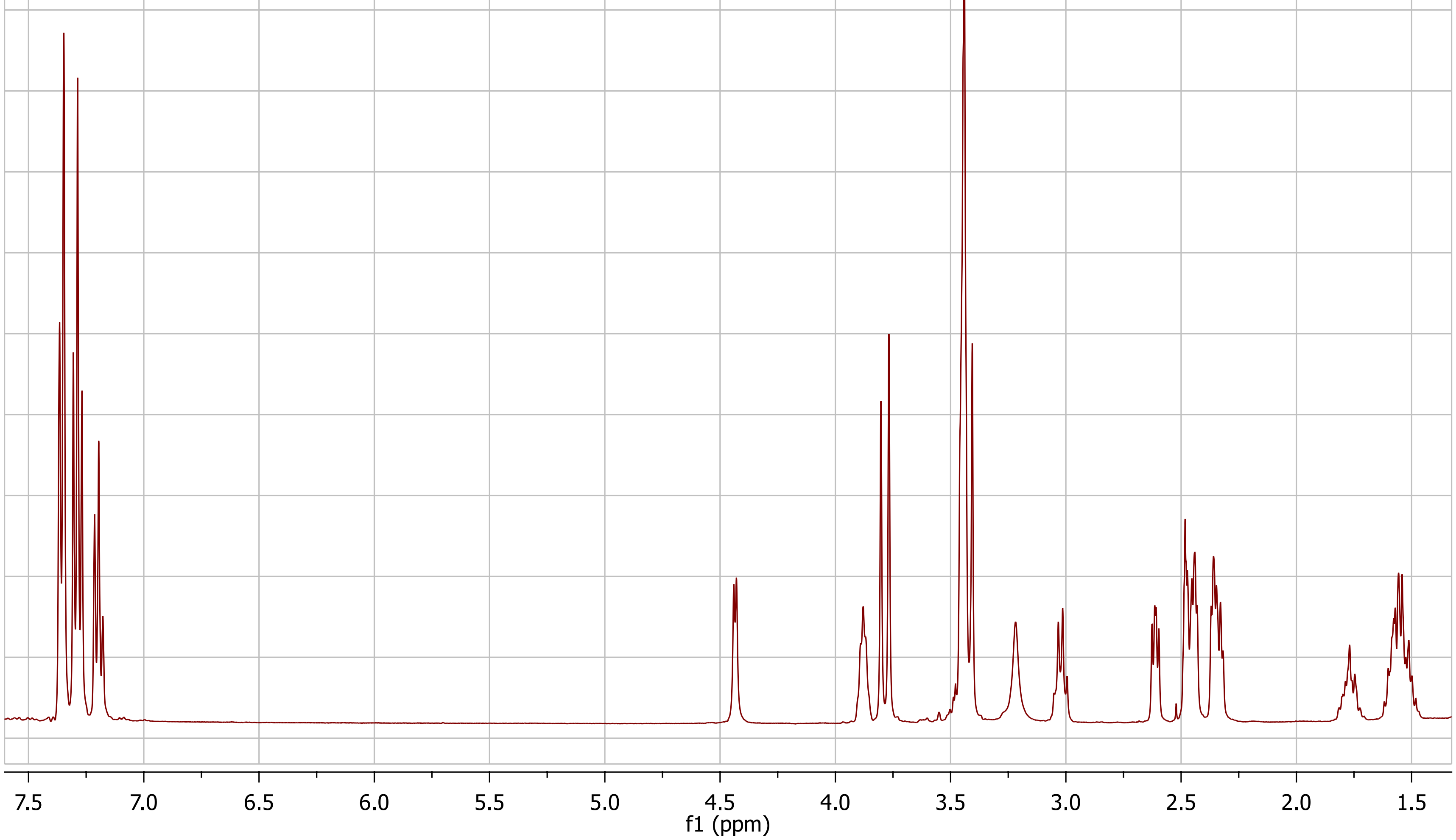


| | Параметр | Значение |
|---|---|---|
| 1 | Origin | Varian |
| 2 | Spectrometer | mercury |
| 3 | Author | vnmr1 |
| 4 | Solvent | DMSO |
| 5 | Temperature | 50.0 |
| 6 | Pulse Sequence | s2pul |
| 7 | Number of Scans | 4 |
| 8 | Receiver Gain | 4 |
| 9 | Relaxation Delay | 3.0000 |
| 10 | Pulse Width | 0.0000 |
| 11 | Acquisition Time | 1.4003 |
| 12 | Gradient Duration | 0.0020 |
| 13 | Spectrometer Frequency | 400.45 |
| 14 | Spectral Width | 2513.8 |
| 15 | Lowest Frequency | 531.3 |
| 16 | Nucleus | 1H |
| 17 | Acquired Size | 3520 |
| 18 | Spectral Size | 8192 |

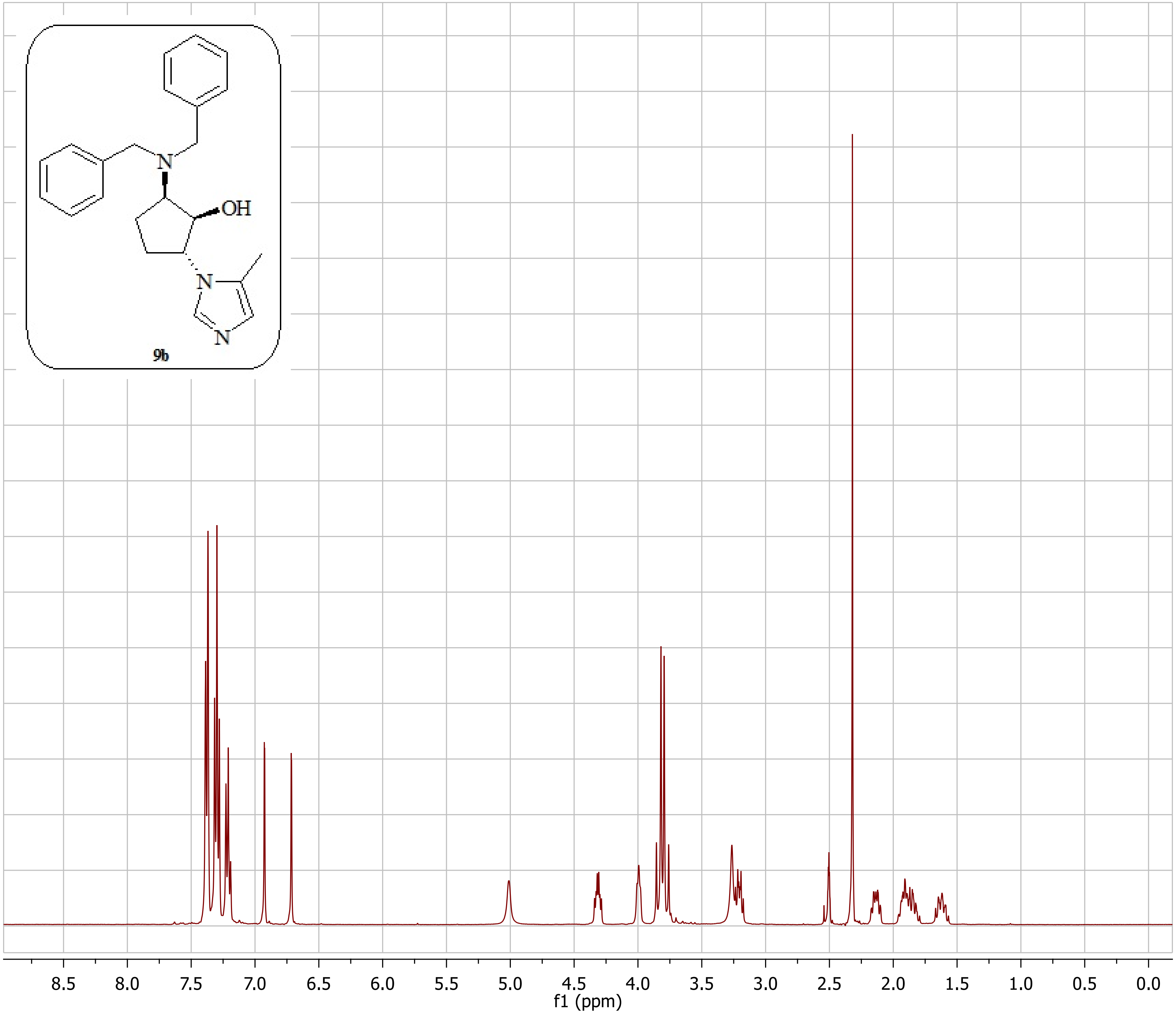


| | Параметр | Значение |
|---|---|---|
| 1 | Origin | Varian |
| 2 | Spectrometer | mercury |
| 3 | Author | vnmr1 |
| 4 | Solvent | DMSO |
| 5 | Temperature | 50.0 |
| 6 | Pulse Sequence | s222 |
| 7 | Number of Scans | 1 |
| 8 | Receiver Gain | 4 |
| 9 | Relaxation Delay | 3.0000 |
| 10 | Pulse Width | 0.0000 |
| 11 | Acquisition Time | 1.4000 |
| 12 | Gradient Duration | 0.0020 |
| 13 | Spectrometer Frequency | 400.46 |
| 14 | Spectral Width | 6535.9 |
| 15 | Lowest Frequency | -376.1 |
| 16 | Nucleus | 1H |
| 17 | Acquired Size | 9150 |
| 18 | Spectral Size | 32768 |

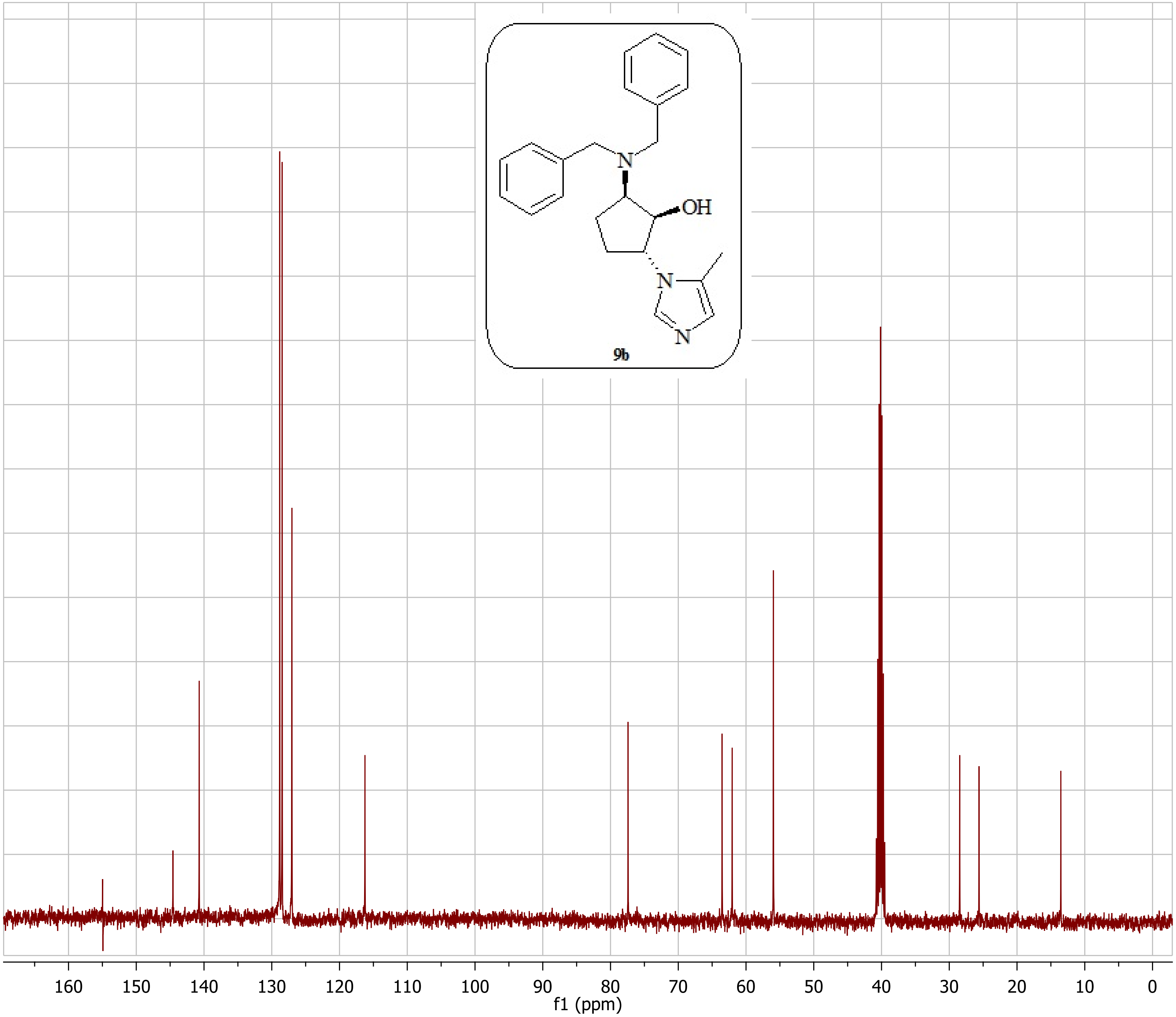


| | Параметр | Значение |
|---|---|---|
| 1 | Origin | Varian |
| 2 | Spectrometer | mercury |
| 3 | Author | vnmr1 |
| 4 | Solvent | dmso |
| 5 | Temperature | 50.0 |
| 6 | Pulse Sequence | s2pul |
| 7 | Number of Scans | 256 |
| 8 | Receiver Gain | 38 |
| 9 | Relaxation Delay | 3.0000 |
| 10 | Pulse Width | 0.0000 |
| 11 | Acquisition Time | 2.4448 |
| 12 | Gradient Duration | 0.0020 |
| 13 | Spectrometer Frequency | 100.71 |
| 14 | Spectral Width | 26178.0 |
| 15 | Lowest Frequency | -1022.5 |
| 16 | Nucleus | 13C |
| 17 | Acquired Size | 64000 |
| 18 | Spectral Size | 131072 |

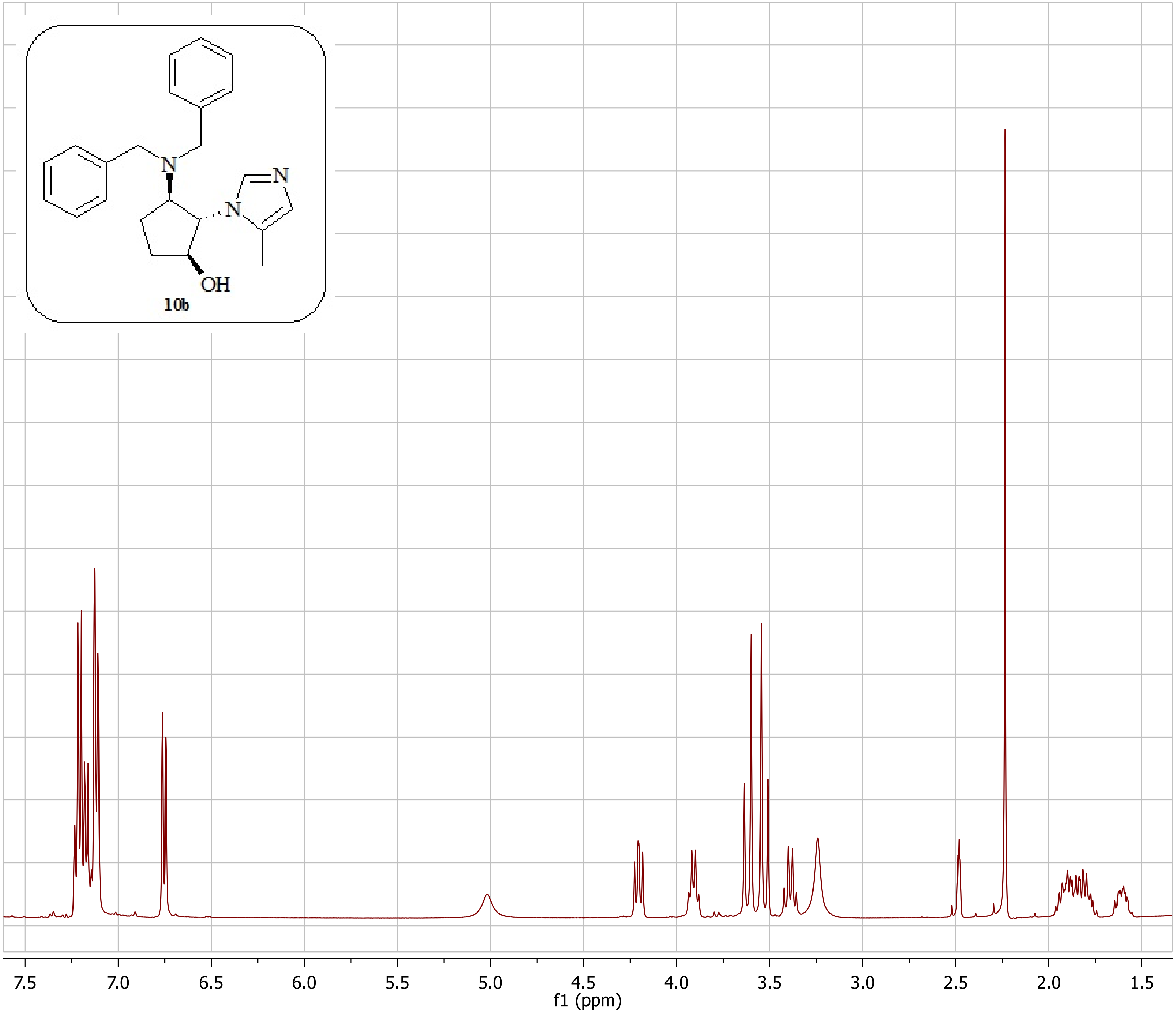


| | Параметр | Значение |
|---|---|---|
| 1 | Origin | Varian |
| 2 | Spectrometer | mercury |
| 3 | Author | vnmr1 |
| 4 | Solvent | DMSO |
| 5 | Temperature | 50.0 |
| 6 | Pulse Sequence | s2pul |
| 7 | Number of Scans | 16 |
| 8 | Receiver Gain | 4 |
| 9 | Relaxation Delay | 3.0000 |
| 10 | Pulse Width | 0.0000 |
| 11 | Acquisition Time | 2.5002 |
| 12 | Gradient Duration | 0.0020 |
| 13 | Spectrometer Frequency | 400.45 |
| 14 | Spectral Width | 2513.8 |
| 15 | Lowest Frequency | 535.7 |
| 16 | Nucleus | 1H |
| 17 | Acquired Size | 6285 |
| 18 | Spectral Size | 16384 |

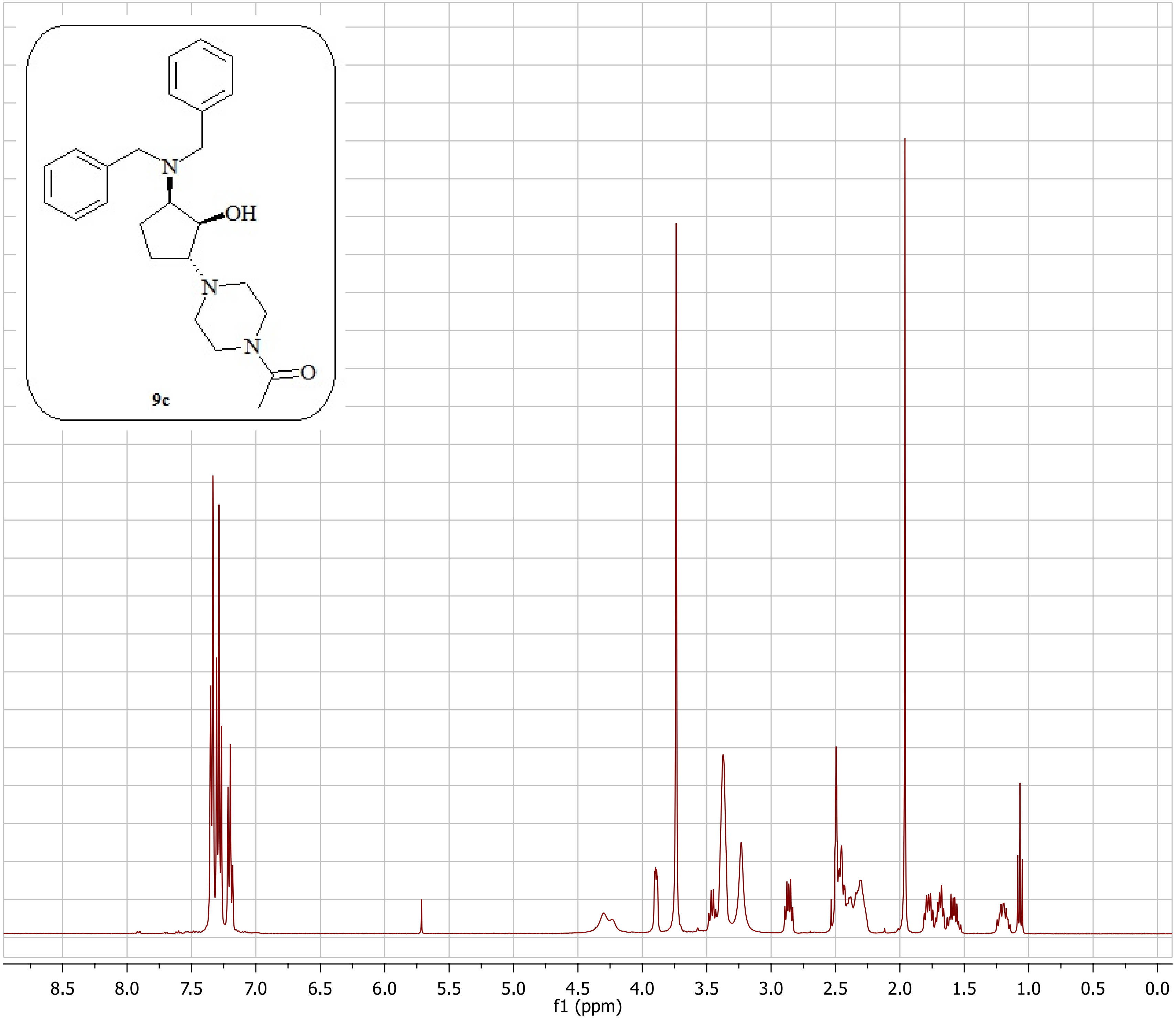


| | Параметр | Значение |
|---|---|---|
| 1 | Origin | Varian |
| 2 | Spectrometer | mercury |
| 3 | Author | vnmr1 |
| 4 | Solvent | DMSO |
| 5 | Temperature | 50.0 |
| 6 | Pulse Sequence | s222 |
| 7 | Number of Scans | 4 |
| 8 | Receiver Gain | 8 |
| 9 | Relaxation Delay | 3.0000 |
| 10 | Pulse Width | 0.0000 |
| 11 | Acquisition Time | 1.4000 |
| 12 | Gradient Duration | 0.0020 |
| 13 | Spectrometer Frequency | 400.46 |
| 14 | Spectral Width | 6535.9 |
| 15 | Lowest Frequency | -379.8 |
| 16 | Nucleus | 1H |
| 17 | Acquired Size | 9150 |
| 18 | Spectral Size | 32768 |

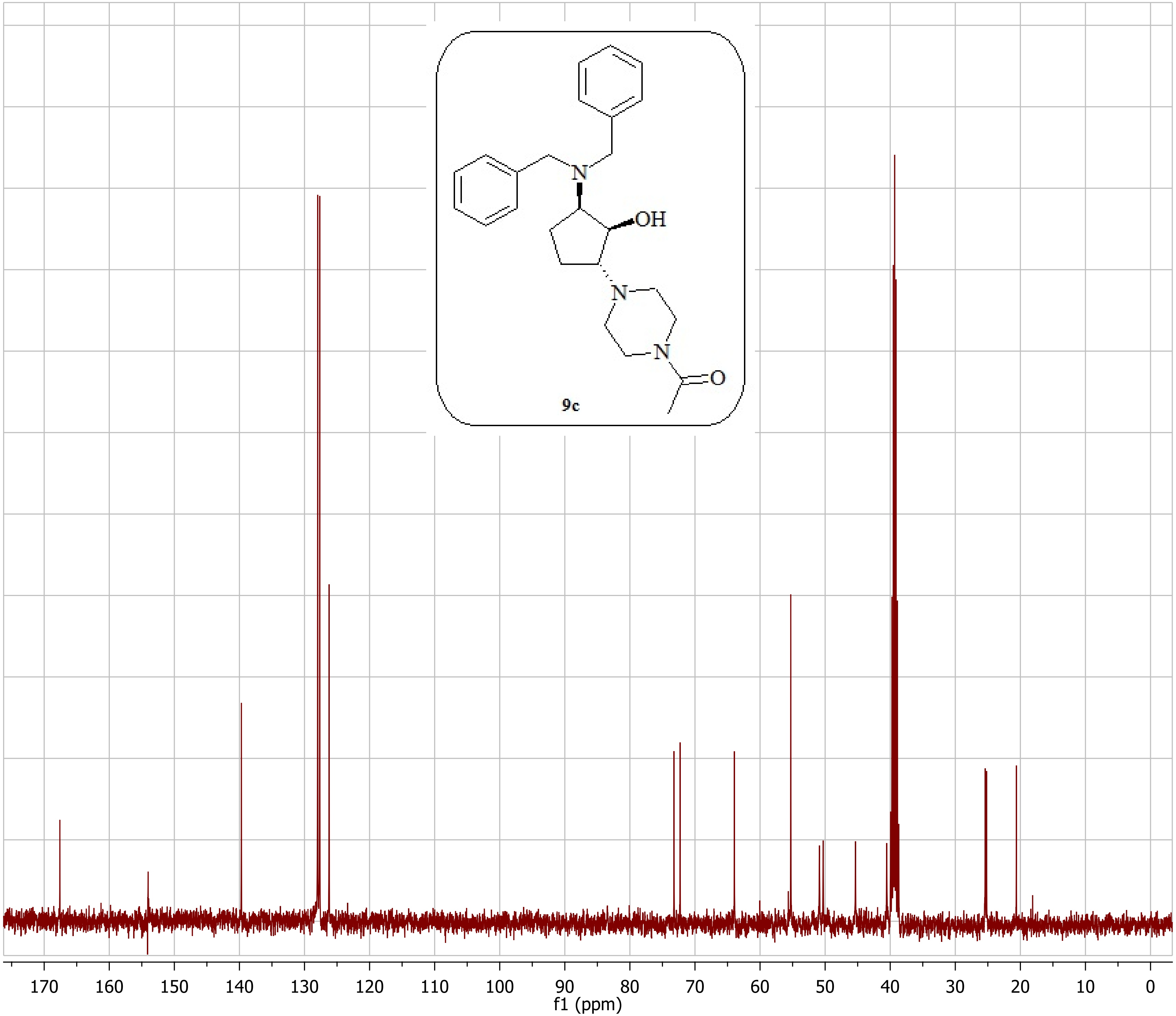


| | Параметр | Значение |
|---|---|---|
| 1 | Origin | Varian |
| 2 | Spectrometer | mercury |
| 3 | Author | vnmr1 |
| 4 | Solvent | dmso |
| 5 | Temperature | 50.0 |
| 6 | Pulse Sequence | s2pul |
| 7 | Number of Scans | 256 |
| 8 | Receiver Gain | 38 |
| 9 | Relaxation Delay | 3.0000 |
| 10 | Pulse Width | 0.0000 |
| 11 | Acquisition Time | 2.4448 |
| 12 | Gradient Duration | 0.0020 |
| 13 | Spectrometer Frequency | 100.71 |
| 14 | Spectral Width | 26178.0 |
| 15 | Lowest Frequency | -1106.4 |
| 16 | Nucleus | 13C |
| 17 | Acquired Size | 64000 |
| 18 | Spectral Size | 131072 |

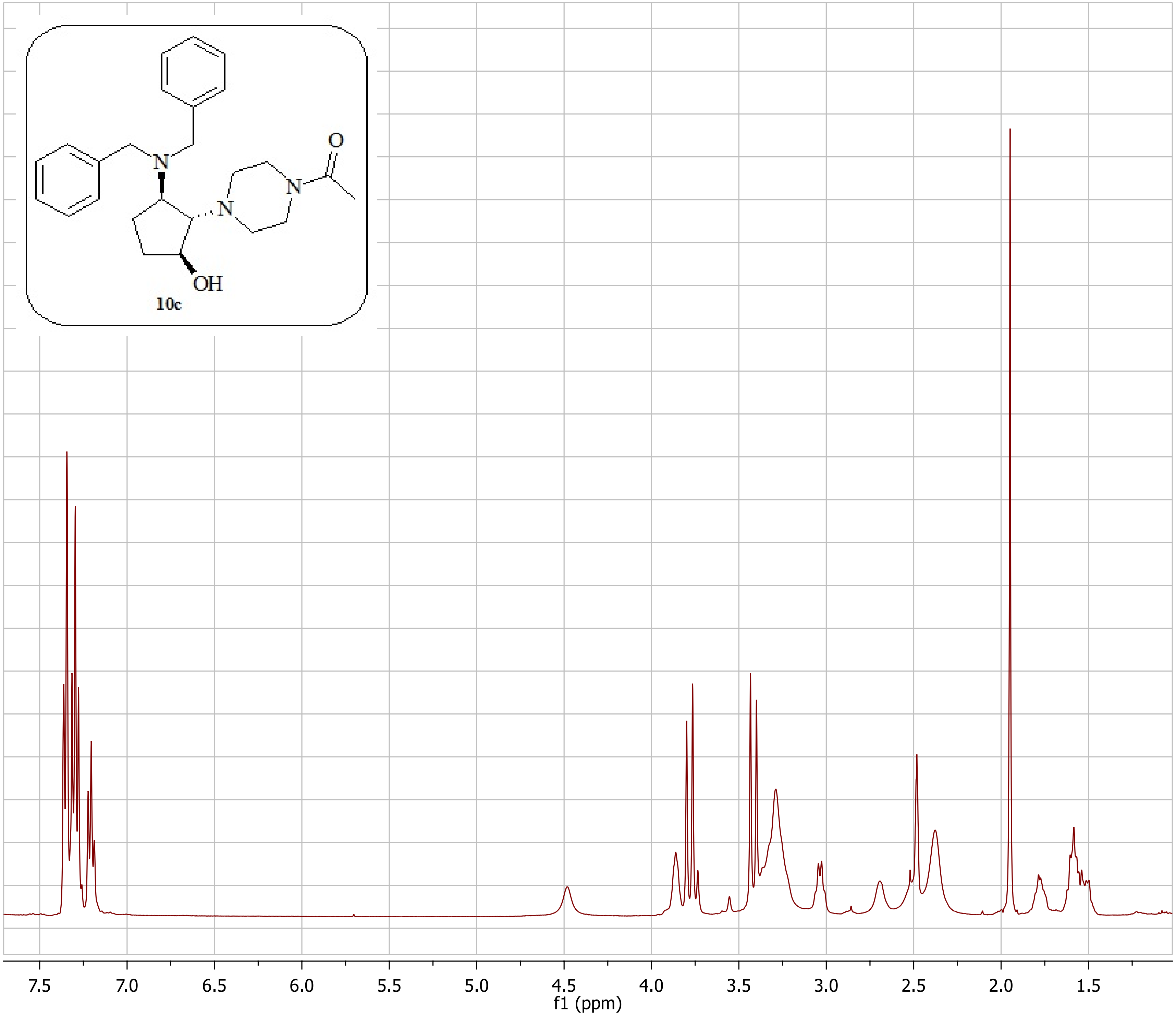


| | Параметр | Значение |
|---|---|---|
| 1 | Origin | Varian |
| 2 | Spectrometer | mercury |
| 3 | Author | vnmr1 |
| 4 | Solvent | DMSO |
| 5 | Temperature | 50.0 |
| 6 | Pulse Sequence | s2pul |
| 7 | Number of Scans | 4 |
| 8 | Receiver Gain | 8 |
| 9 | Relaxation Delay | 3.0000 |
| 10 | Pulse Width | 0.0000 |
| 11 | Acquisition Time | 1.3991 |
| 12 | Gradient Duration | 0.0020 |
| 13 | Spectrometer Frequency | 400.45 |
| 14 | Spectral Width | 2677.4 |
| 15 | Lowest Frequency | 408.6 |
| 16 | Nucleus | 1H |
| 17 | Acquired Size | 3746 |
| 18 | Spectral Size | 8192 |

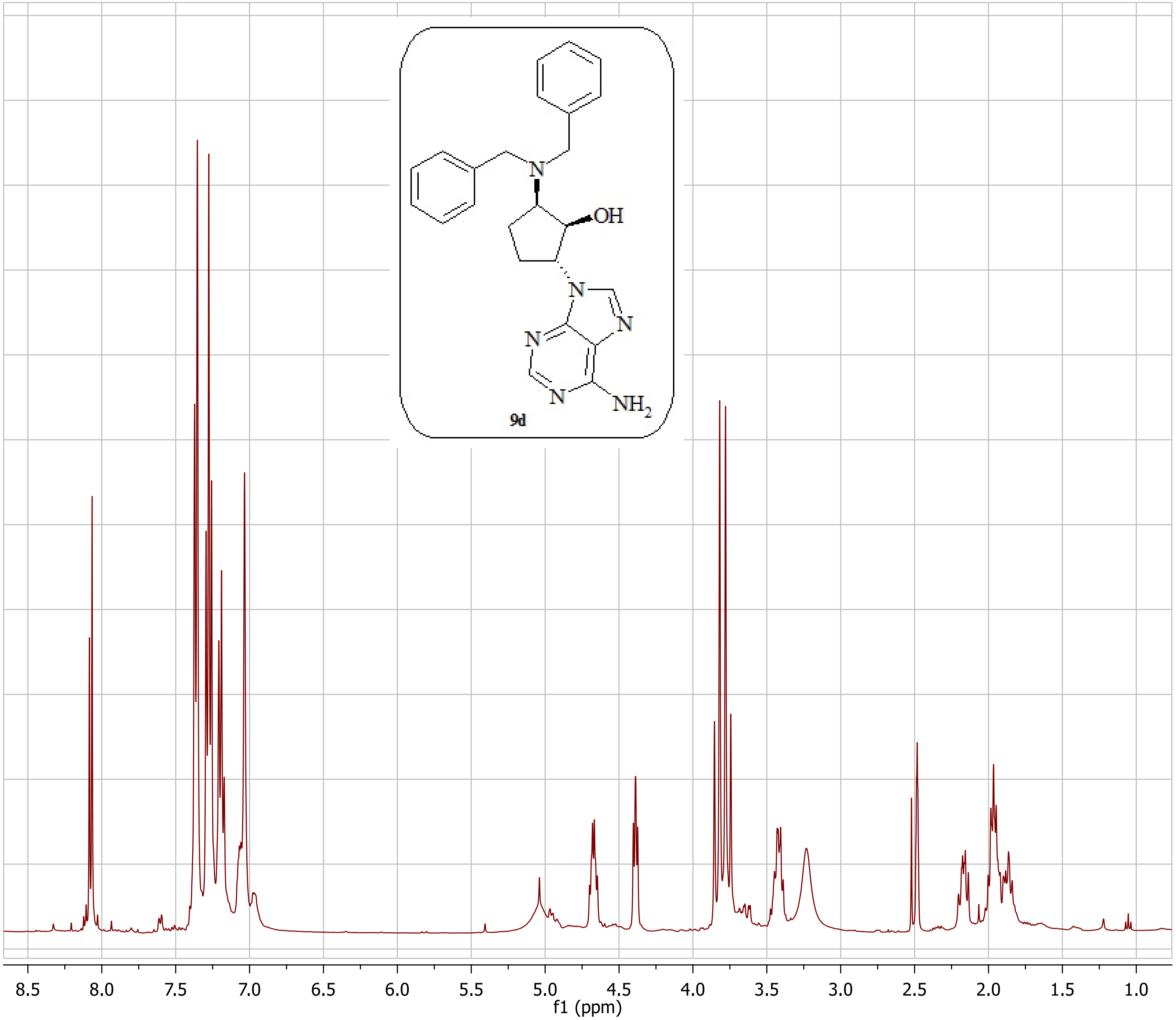


| | Параметр | Значение |
|---|---|---|
| 1 | Origin | Varian |
| 2 | Spectrometer | mercury |
| 3 | Author | vnmr1 |
| 4 | Solvent | DMSO |
| 5 | Temperature | 50.0 |
| 6 | Pulse Sequence | s2pul |
| 7 | Number of Scans | 16 |
| 8 | Receiver Gain | 8 |
| 9 | Relaxation Delay | 3.0000 |
| 10 | Pulse Width | 0.0000 |
| 11 | Acquisition Time | 2.5000 |
| 12 | Gradient Duration | 0.0020 |
| 13 | Spectrometer Frequency | 400.45 |
| 14 | Spectral Width | 3165.6 |
| 15 | Lowest Frequency | 304.0 |
| 16 | Nucleus | 1H |
| 17 | Acquired Size | 7914 |
| 18 | Spectral Size | 16384 |

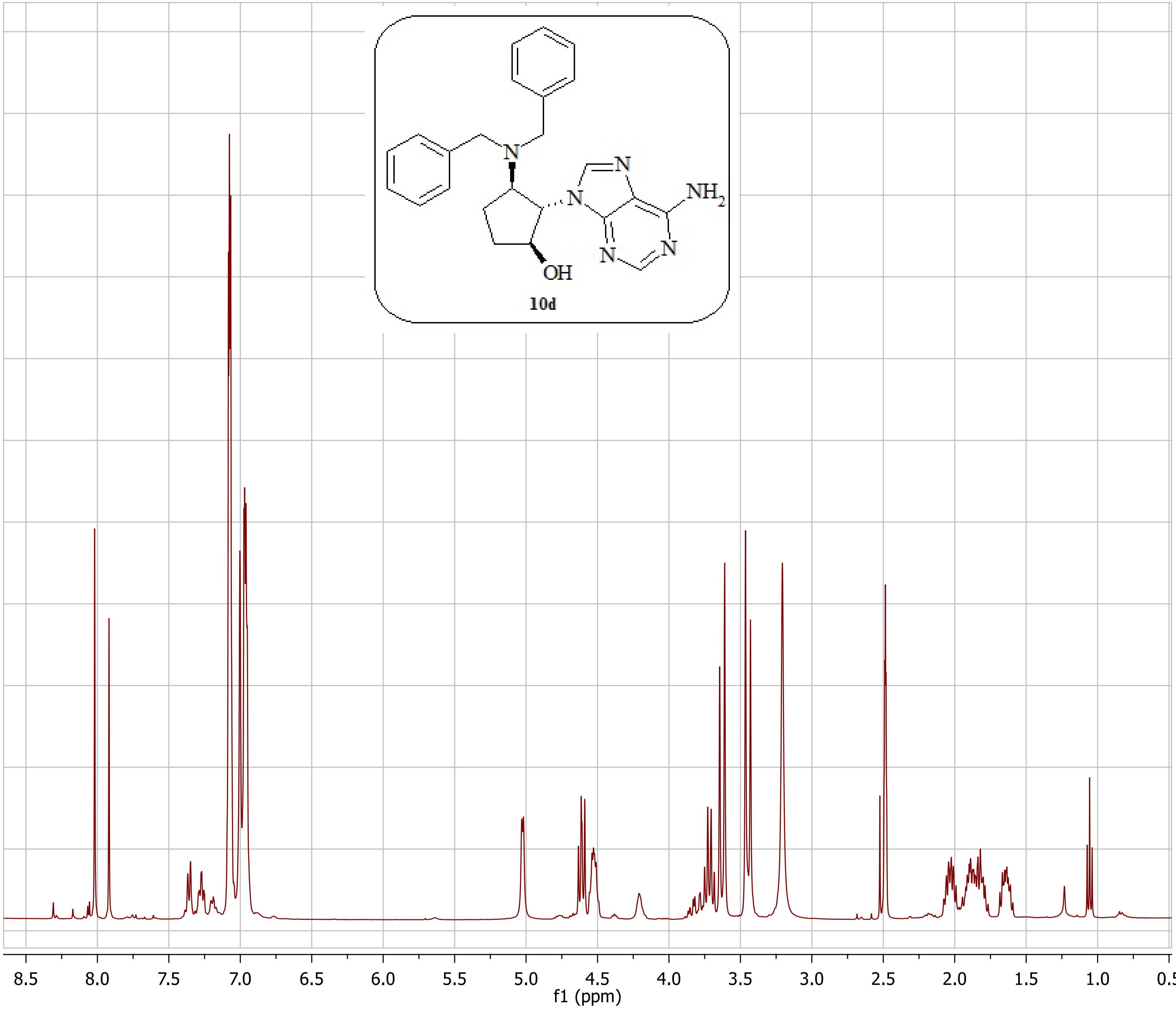


| | Параметр | Значение |
|---|---|---|
| 1 | Origin | Varian |
| 2 | Spectrometer | mercury |
| 3 | Author | vnmr1 |
| 4 | Solvent | DMSO |
| 5 | Temperature | 50.0 |
| 6 | Pulse Sequence | s2pul |
| 7 | Number of Scans | 16 |
| 8 | Receiver Gain | 12 |
| 9 | Relaxation Delay | 3.0000 |
| 10 | Pulse Width | 0.0000 |
| 11 | Acquisition Time | 2.4999 |
| 12 | Gradient Duration | 0.0020 |
| 13 | Spectrometer Frequency | 400.45 |
| 14 | Spectral Width | 3273.3 |
| 15 | Lowest Frequency | 193.2 |
| 16 | Nucleus | 1H |
| 17 | Acquired Size | 8183 |
| 18 | Spectral Size | 16384 |

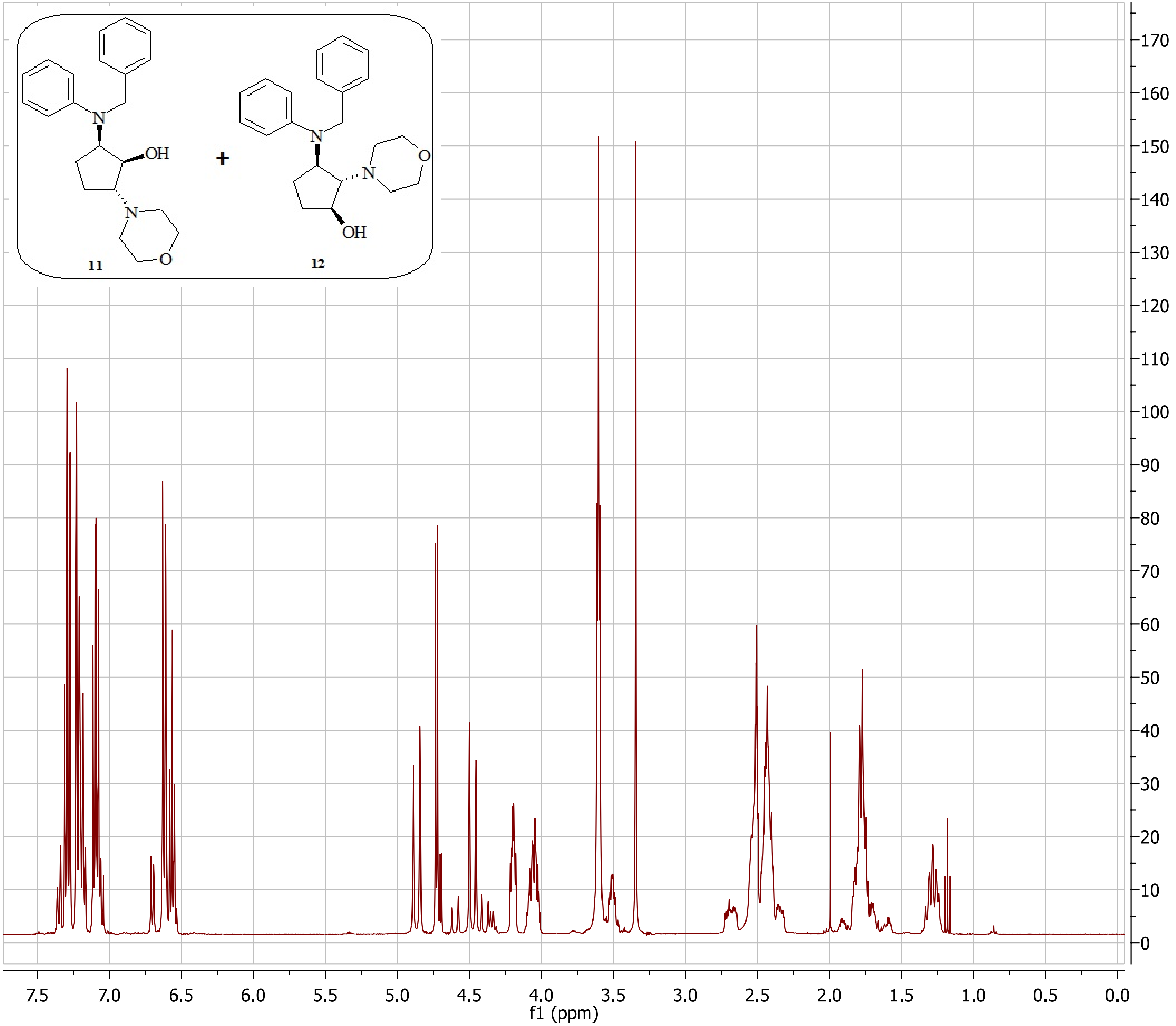


| | Параметр | Значение |
|---|---|---|
| 1 | Origin | Varian |
| 2 | Spectrometer | vnmrs |
| 3 | Author | chemp |
| 4 | Solvent | dmso |
| 5 | Temperature | 34.0 |
| 6 | Pulse Sequence | s2pul |
| 7 | Number of Scans | 16 |
| 8 | Receiver Gain | 30 |
| 9 | Relaxation Delay | 1.0000 |
| 10 | Pulse Width | 0.0000 |
| 11 | Acquisition Time | 2.5559 |
| 12 | Gradient Duration | 0.0010 |
| 13 | Spectrometer Frequency | 399.70 |
| 14 | Spectral Width | 6410.3 |
| 15 | Lowest Frequency | -784.9 |
| 16 | Nucleus | 1H |
| 17 | Acquired Size | 16384 |
| 18 | Spectral Size | 32768 |

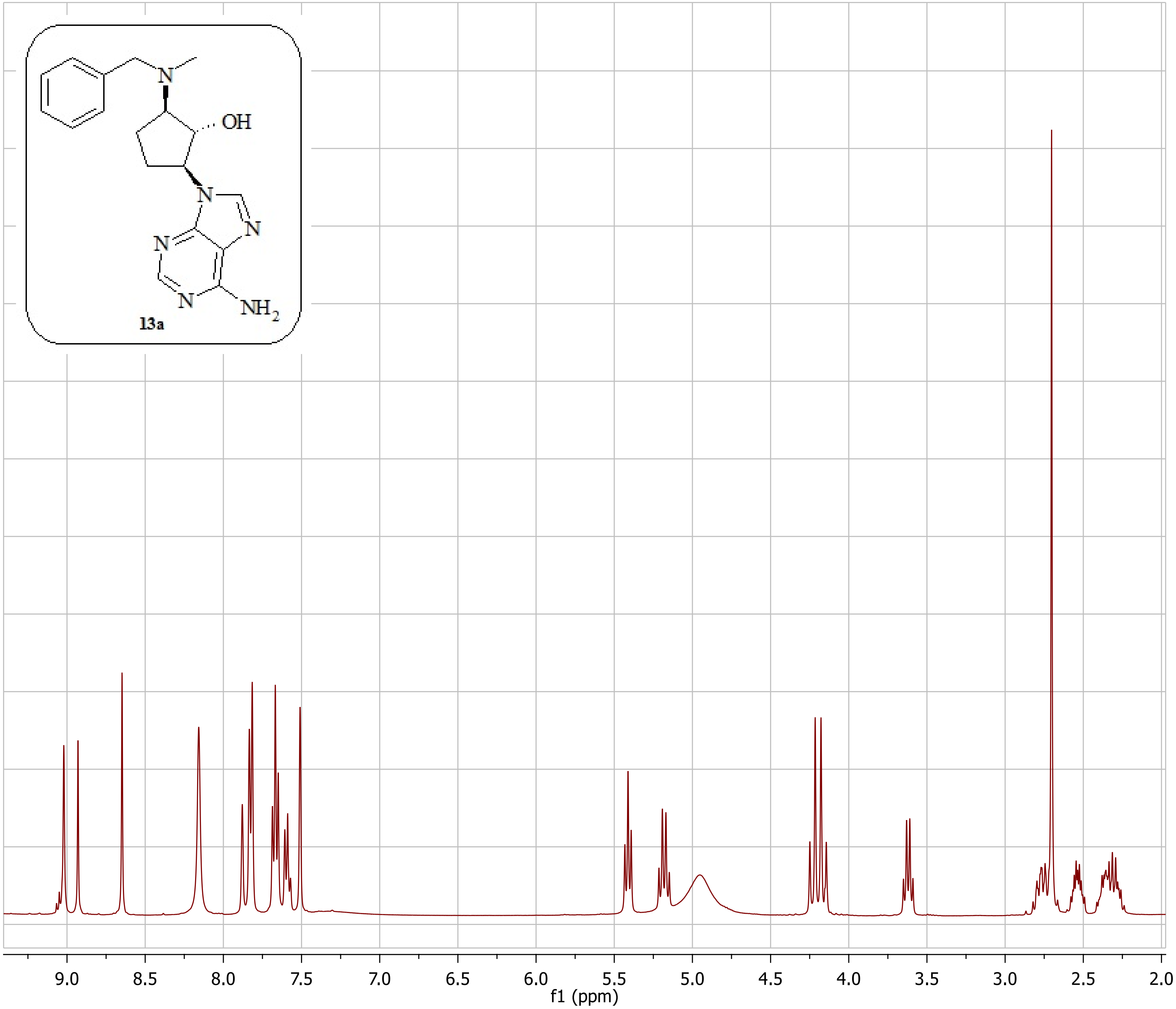


| | Параметр | Значение |
|---|---|---|
| 1 | Origin | Varian |
| 2 | Spectrometer | mercury |
| 3 | Author | vnmr1 |
| 4 | Solvent | pyridine |
| 5 | Temperature | 50.0 |
| 6 | Pulse Sequence | s2pul |
| 7 | Number of Scans | 16 |
| 8 | Receiver Gain | 16 |
| 9 | Relaxation Delay | 3.0000 |
| 10 | Pulse Width | 0.0000 |
| 11 | Acquisition Time | 2.4998 |
| 12 | Gradient Duration | 0.0020 |
| 13 | Spectrometer Frequency | 400.45 |
| 14 | Spectral Width | 2976.2 |
| 15 | Lowest Frequency | 790.3 |
| 16 | Nucleus | 1H |
| 17 | Acquired Size | 7440 |
| 18 | Spectral Size | 16384 |

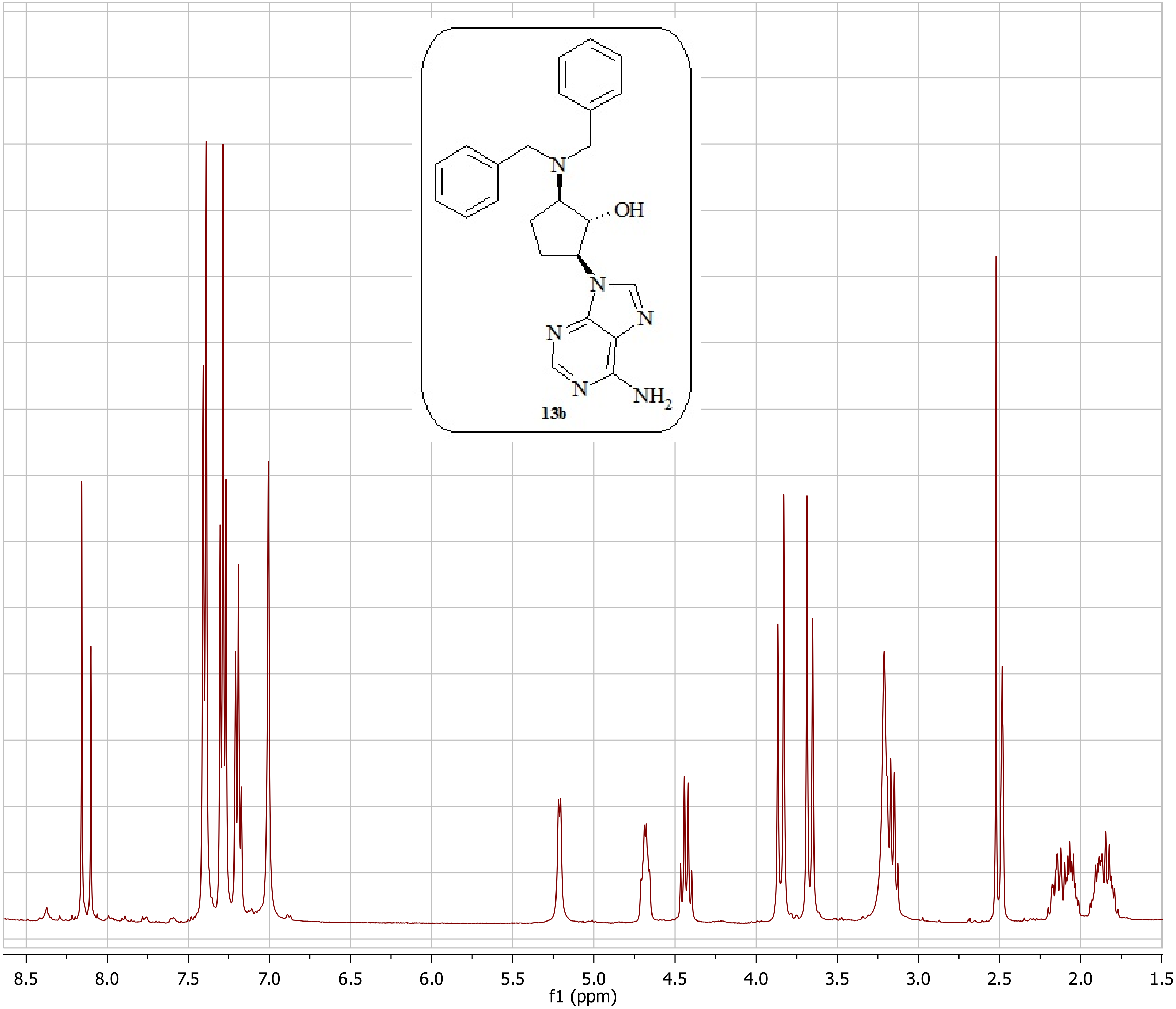


| | Параметр | Значение |
|---|---|---|
| 1 | Origin | Varian |
| 2 | Spectrometer | mercury |
| 3 | Author | vnmr1 |
| 4 | Solvent | DMSO |
| 5 | Temperature | 50.0 |
| 6 | Pulse Sequence | s2pul |
| 7 | Number of Scans | 16 |
| 8 | Receiver Gain | 8 |
| 9 | Relaxation Delay | 3.0000 |
| 10 | Pulse Width | 0.0000 |
| 11 | Acquisition Time | 2.5000 |
| 12 | Gradient Duration | 0.0020 |
| 13 | Spectrometer Frequency | 400.45 |
| 14 | Spectral Width | 2861.2 |
| 15 | Lowest Frequency | 598.0 |
| 16 | Nucleus | 1H |
| 17 | Acquired Size | 7153 |
| 18 | Spectral Size | 16384 |

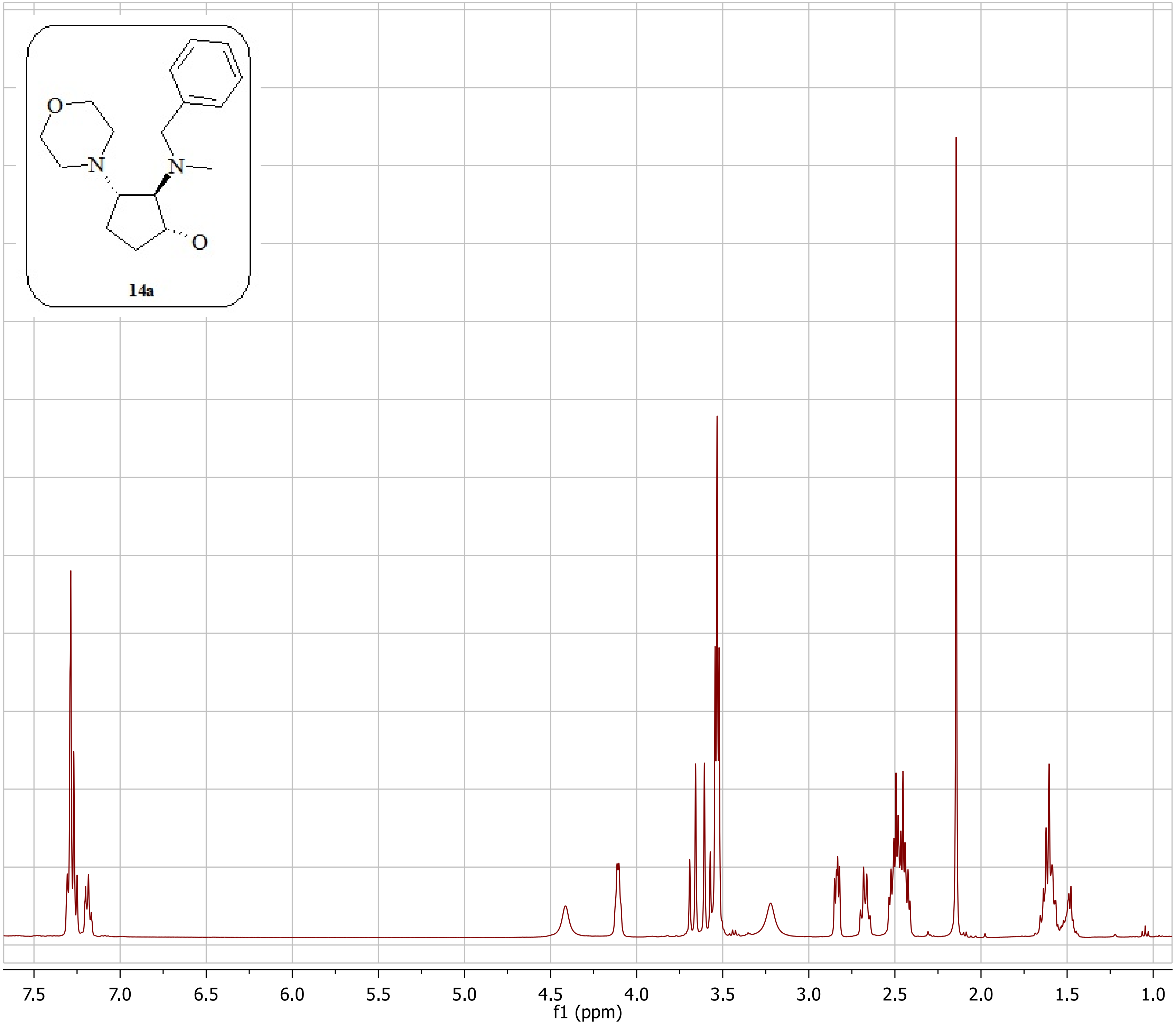


| | Параметр | Значение |
|---|---|---|
| 1 | Origin | Varian |
| 2 | Spectrometer | mercury |
| 3 | Author | vnmr1 |
| 4 | Solvent | DMSO |
| 5 | Temperature | 50.0 |
| 6 | Pulse Sequence | s2pul |
| 7 | Number of Scans | 16 |
| 8 | Receiver Gain | 6 |
| 9 | Relaxation Delay | 3.0000 |
| 10 | Pulse Width | 0.0000 |
| 11 | Acquisition Time | 2.5035 |
| 12 | Gradient Duration | 0.0020 |
| 13 | Spectrometer Frequency | 400.45 |
| 14 | Spectral Width | 2717.4 |
| 15 | Lowest Frequency | 356.6 |
| 16 | Nucleus | 1H |
| 17 | Acquired Size | 6803 |
| 18 | Spectral Size | 16384 |

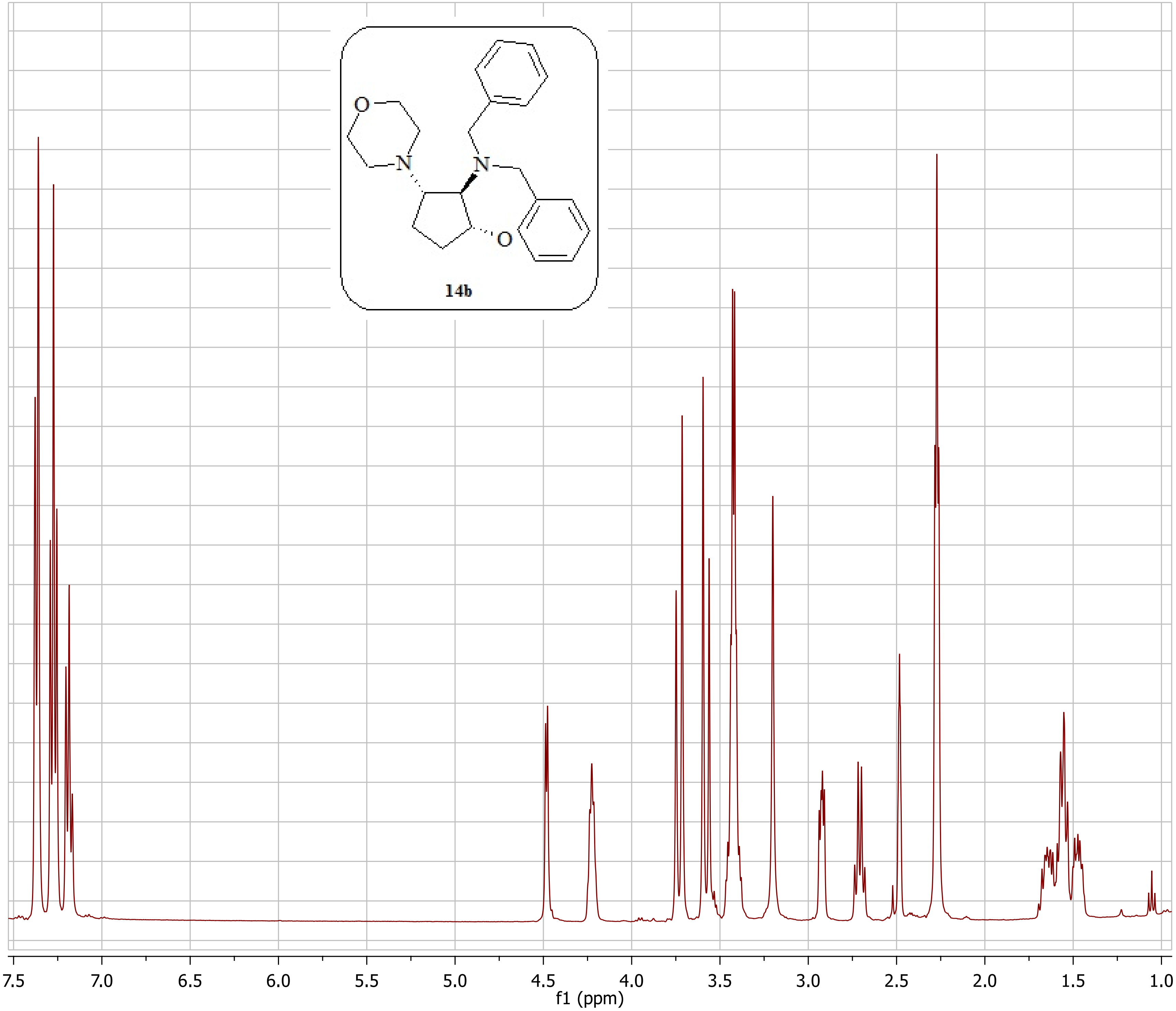


| | Параметр | Значение |
|---|---|---|
| 1 | Origin | Varian |
| 2 | Spectrometer | mercury |
| 3 | Author | vnmr1 |
| 4 | Solvent | DMSO |
| 5 | Temperature | 50.0 |
| 6 | Pulse Sequence | s2pul |
| 7 | Number of Scans | 4 |
| 8 | Receiver Gain | 8 |
| 9 | Relaxation Delay | 3.0000 |
| 10 | Pulse Width | 0.0000 |
| 11 | Acquisition Time | 1.3996 |
| 12 | Gradient Duration | 0.0020 |
| 13 | Spectrometer Frequency | 400.45 |
| 14 | Spectral Width | 2637.8 |
| 15 | Lowest Frequency | 377.3 |
| 16 | Nucleus | 1H |
| 17 | Acquired Size | 3692 |
| 18 | Spectral Size | 8192 |

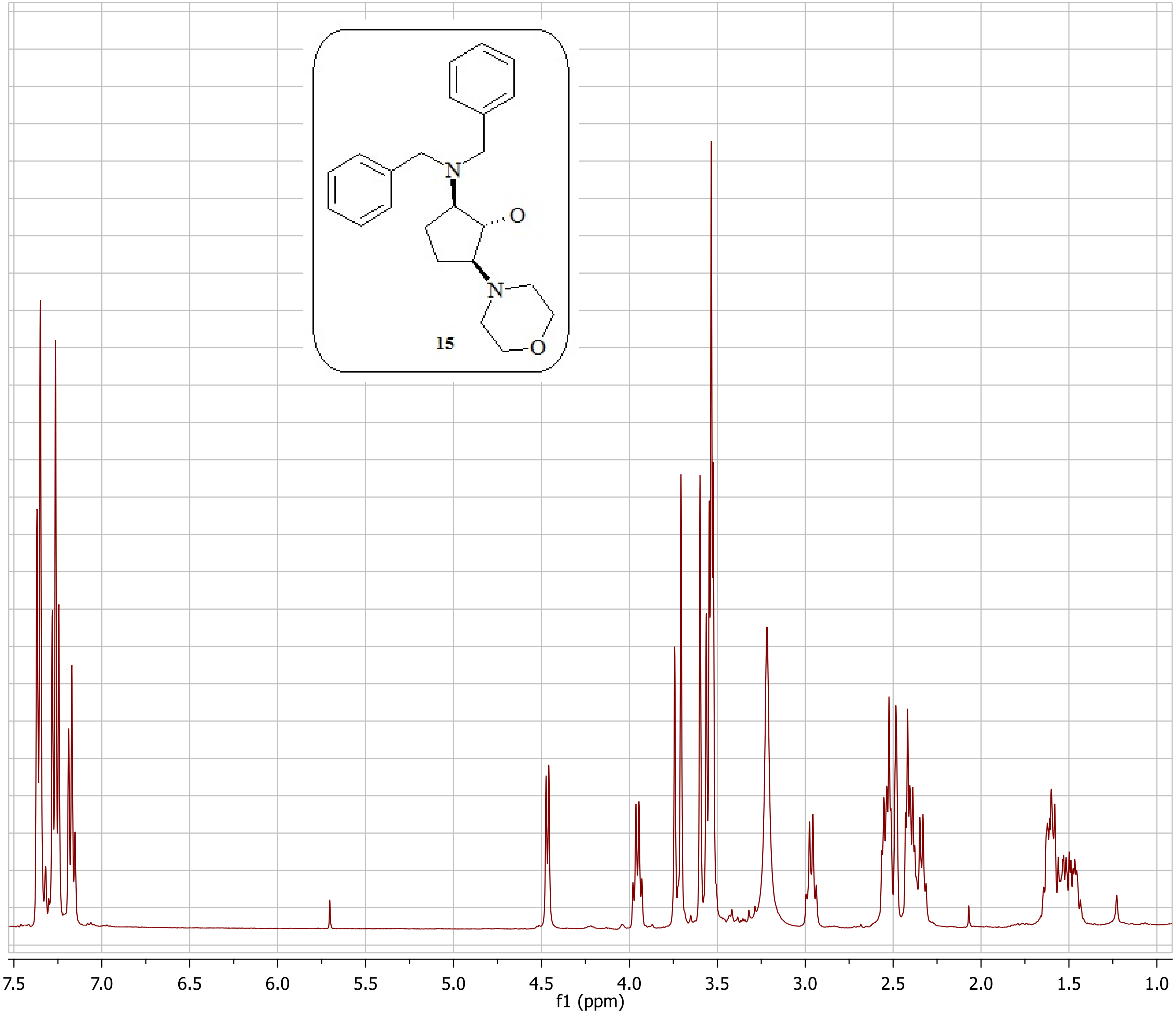


| | Параметр | Значение |
|---|---|---|
| 1 | Origin | Varian |
| 2 | Spectrometer | mercury |
| 3 | Author | vnmr1 |
| 4 | Solvent | DMSO |
| 5 | Temperature | 50.0 |
| 6 | Pulse Sequence | s2pul |
| 7 | Number of Scans | 4 |
| 8 | Receiver Gain | 8 |
| 9 | Relaxation Delay | 3.0000 |
| 10 | Pulse Width | 0.0000 |
| 11 | Acquisition Time | 1.4007 |
| 12 | Gradient Duration | 0.0020 |
| 13 | Spectrometer Frequency | 400.45 |
| 14 | Spectral Width | 2732.2 |
| 15 | Lowest Frequency | 286.3 |
| 16 | Nucleus | 1H |
| 17 | Acquired Size | 3827 |
| 18 | Spectral Size | 8192 |
